# 3D Affine: An Embedding of Local Image Features for Viewpoint Invariance Using RGB-D Sensor Data

**DOI:** 10.3390/s19020291

**Published:** 2019-01-12

**Authors:** Hamdi Sahloul, Shouhei Shirafuji, Jun Ota

**Affiliations:** 1 Department of Precision Engineering, Graduate School of Engineering, The University of Tokyo, 7-3-1 Hongo, Bunkyo-ku, Tokyo 113-8656, Japan; 2 Human-Artifactology Research Division, Research into Artifacts, Center for Engineering (RACE), The University of Tokyo, 5-1-5 Kashiwanoha, Kashiwa-shi, Chiba 277-8568, Japan; shirafuji@race.u-tokyo.ac.jp (S.S.); ota@race.u-tokyo.ac.jp (J.O.)

**Keywords:** viewpoint invariance, local image feature embedding, wide baseline matching, out-of-plane rotations, 6D pose estimation, nonparametric spherical k-means, denoising and interpolation, 3D points projection

## Abstract

Local image features are invariant to in-plane rotations and robust to minor viewpoint changes. However, the current detectors and descriptors for local image features fail to accommodate out-of-plane rotations larger than 25°–30°. Invariance to such viewpoint changes is essential for numerous applications, including wide baseline matching, 6D pose estimation, and object reconstruction. In this study, we present a general embedding that wraps a detector/descriptor pair in order to increase viewpoint invariance by exploiting input depth maps. The proposed embedding locates smooth surfaces within the input RGB-D images and projects them into a viewpoint invariant representation, enabling the detection and description of more viewpoint invariant features. Our embedding can be utilized with different combinations of descriptor/detector pairs, according to the desired application. Using synthetic and real-world objects, we evaluated the viewpoint invariance of various detectors and descriptors, for both *standalone* and *embedded* approaches. While standalone local image features fail to accommodate average viewpoint changes beyond 33.3°, our proposed embedding boosted the viewpoint invariance to different levels, depending on the scene geometry. Objects with distinct surface discontinuities were on average invariant up to 52.8°, and the overall average for all evaluated datasets was 45.4°. Similarly, out of a total of 140 combinations involving 20 local image features and various objects with distinct surface discontinuities, only a single standalone local image feature exceeded the goal of 60° viewpoint difference in just two combinations, as compared with 19 different local image features succeeding in 73 combinations when wrapped in the proposed embedding. Furthermore, the proposed approach operates robustly in the presence of input depth noise, even that of low-cost commodity depth sensors, and well beyond.

## 1. Introduction

Local image features are major low-level building blocks in various computer vision and image processing algorithms; however, they have a certain degree of sensitivity to viewpoint changes. In light of the recent growth in the applications of RGB-D sensors, the viewpoint invariance of local image features has been gradually improving [1,2,3,4] by utilizing input depth maps. Despite the current trend of using machine learning for low-level local image features [5,6,7,8,9,10,11], or even for high-level tasks, such as 6D pose estimation, [12,13,14,15,16], several hand-crafted features are still actively employed in various algorithms. This trend is due to the on-par or better performance of hand-crafted features [17] and their well-established maturity gained from 10–20 years of research. Examples of hand-crafted features include GFTT (Good Features to Track) [18], SIFT (Scale-Invariant Feature Transform) [19], and SURF (Speeded Up Robust Features) [20]. Nevertheless, these are local intensity-image approaches, which typically *detect* keypoints and then *describe* their features from image patches (hence the name *local*) with gray or trichromatic intensities *under some geometric assumptions*. Thus, they tend to lack robustness to geometric transformations involving mainly viewpoint changes, being robust up to 25°–30° [21]. Consequently, issues such as *unrepeatable detection*, *indistinct description*, or *non-covariant keypoints* can arise. These issues essentially originate from the neighborhood perspective changes that accompany out-of-plane rotations, sampling window overlap of a keypoint located near the edges of a surface, or when the surface has different orientation from the viewpoint, respectively. Evidently, Vedaldi and Soatto demonstrated that local image features can achieve viewpoint invariance for generic non-planar scenes without assuming a locally planar scene [22]. Achieving viewpoint invariance will improve the efficiency and robustness of various computer vision *ill-posed problems*, including wide baseline matching, 6D pose estimation (i.e., *rigid body transformation*), 3D reconstruction, recognition by reconstruction, and visual SLAM (Simultaneous Localization And Mapping).

Little research has focused on using geometric information to improve viewpoint invariance. For example, Wu et al. proposed a SIFT-based descriptor that utilizes depth maps to construct VIPs (Viewpoint Invariant Patches) [1]. Similar proposed solutions include a SIFT-based detector with a region-affine sampling window [2], and BRISK (Binary Robust Invariant Scalable Keypoints)-based features with adaptive sampling pattern orientations and scale factors [3,4]. However, many such local image detectors and descriptors are intended for a particular application [23], and the cited studies targeted specific local image features, thus only covering a small subset of the numerous applications presented by each of the vastly available features.

Several studies have presented general *embeddings*. An embedding wraps around local image features, independent of their implementation, for the purpose of enhancing certain aspects of the wrapped features, such as distinctiveness [24,25] or invariance. Specific examples include ASIFT (2D Affine SIFT) [26] and ASURF (2D Affine SURF) [27], which are local image feature embeddings for 2D affinity invariance and are capable of wrapping features in addition to those described in their original SIFT- and SURF-based proposals. While these embeddings do not succumb to the pitfall of targeting specific applications, existing embeddings depend solely on intensity images and are thus limited to in-plane motion invariance, i.e., the *2D-affine transformations*.

We are currently unaware of any available 3D-affine embedding, for which there is a growing need, as the list of local image features continues to grow [5,6,18,19,20,24,25,26,27,28,29,30,31,32,33,34,35,36,37,38,39,40], each being tailored for a particular application and having inadequate viewpoint invariance [21]. To this end, we propose a general 3D-affine embedding that wraps virtually any chosen *standalone* detector/descriptor pair to improve its viewpoint invariance without changing the *invariance formula(s)* or implementation. The aim is not to propose another detector/descriptor, but rather to fill in the gaps and improve existing local image features by enhancing their viewpoint invariance. Briefly, the proposed method aims to achieve viewpoint invariance by: (1) extracting surfaces of similar properties (i.e., *smooth surfaces*) from the scene; (2) representing these surfaces in a *reference local plane* with a viewpoint invariant representation; (3) applying the wrapped detector/descriptor to the viewpoint invariant representation; and (4) mapping the extracted features back to their original input frame, i.e., the *local frame*. This scheme is supposed to address the robustness issues to geometric transformations, namely unrepeatable detection, indistinct description, and non-covariant keypoints. Ideally, the scheme will result in repeatable detection of near or faraway regions, since each smooth surface is brought to the same reference local plane. Similarly, the description overlap between one surface and another is mitigated because feature detection and description are performed independently per each smooth surface. Finally, features will have an affine-region sampling window that will covariantly change with plane orientation, since they are first computed in the reference local plane and then mapped back to their original frame.

Accordingly, the challenges are to achieve a stable viewpoint invariant representation and to provide a general embedding that is applicable to any local image detector/descriptor. Our approach to viewpoint invariant representation involves labeling stable smooth surfaces by back-projecting surface-normal clusters that are aggregated using nonparametric spherical *k*-means (Section 2.2), estimating their warp transforms (Section 2.3), and then warping the labeled surfaces to a reference local plane using a hybrid rigid-homography method (Section 2.4). The generality of the embedding is guaranteed by introducing preprocessing and post-processing stages, before and after the detection/description processes (Section 2.5), to match their standard interfaces. The aforementioned approach forms the preprocessing, and post-processing is to put together all obtained features from the per-surface independent computations by mapping the extracted features to the local frame (Section 2.6). Our main contribution is the development of a general embedding that:

- Improves the viewpoint invariance of local image features,
- Wraps virtually any local image detector/descriptor algorithm, and
- Requires no additional interfacing or modifications.

The remainder of this manuscript is organized as follows. Section 2 describes the proposed embedding, Section 3 demonstrates an application of 6D pose estimation, and Section 4 explains the experimental setup, the datasets, and the performance metrics that were utilized. The results are discussed in Section 5, and Section 6 presents conclusions and future work.

## 2. The Proposed 3D-Affine Embedding Approach

**Figure 1:**
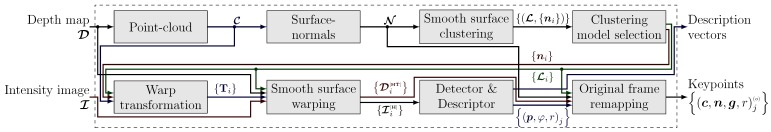
An overview of the proposed 3D-affine embedding pipeline. First, a point-cloud, C, and the surface normals, N, are computed from the input depth map, D (Section 2.1). Then, the surface normals are used to form several spherical *k*-mean modules in parallel, and the best model, $\left(L,\left\{n_{i}\right\}\right)$, is selected to annotate the smooth surfaces in the scene (Section 2.2). After that, all smooth surfaces are parameterized by their point-cloud and surface-normal centroids, $\left\{{\left(c,n\right)}_{i}\right\}$, and their individual warp transforms, $\left\{T_{i}\right\}$, are computed (Section 2.3). Using their corresponding warp transforms, the smooth surfaces are rigidly warped and homographically morphed in parallel into a viewpoint invariant representation, $\left\{\left(I_{i}^{\left[H\right]},D_{i}^{\left[H\tilde{T}\right]}\right)\right\}$ (Section 2.4). Using the embedded detector/descriptor, keypoints are detected and then described in parallel from the intensity components of the invariant surfaces, $\left\{I_{i}^{\left[H\right]}\right\}$ (Section 2.5). Finally, the depth components of the smooth surfaces, $\left\{D_{i}^{\left[H\tilde{T}\right]}\right\}$, are used to remap the coordinates back to the RGB-D input frame in parallel, by inverting the warp transforms on the extracted keypoints, ${\left(p,\varphi ,r\right)}_{j}$, and augmenting them with more information about the 3D space, ${\left(c,n,g,r\right)}_{j}^{\left(o\right)}$ (Section 2.6). For a summary of notations, see Table 1.

Our proposed method follows the standard *two-steps scheme*: keypoint detection and feature description from an intensity-image, with pre- and post- processing stages. The preprocessing stage comprises smooth-surface annotation (Section 2.2), warp transform estimation (Section 2.3), and smooth-surface viewpoint invariant representation (Section 2.4). After preprocessing, the features are computed using the local image detector/descriptor pair of choice (Section 2.5). Finally, the post-processing stage puts together all the scene features, which are independently extracted from different surfaces, by mapping their corresponding keypoints back to the local frame of the original input (Section 2.6), which also provides the appropriate affine-region keypoint representation. See Figure 1 for an overview of our approach, and refer to Table 1 for a summary of the notation we use throughout this manuscript.

Our proposed method offers both 3D-affine invariance, similar to VIPs [1], and embedding flexibility (e.g., [26,27]) that enables wrapping any local image detector/descriptor approach that follows the two-steps scheme. For VIPs, detection is performed before warping, while description comes after it, thus might not address repeatability issues. Per contra, we allow both detection and description from the warped surfaces, by applying smooth-surface labeling using a nonparametric method based on spherical *k*-means [41], as described in Section 2.2. Section 2.4 describes a similar approach to VIPs warping, which we further enhanced to generate flawless and accurate representations, by proposing a hybrid rigid-homography warping (Section 2.4.3). Section 2.5 and Section 2.6 describe detecting, extracting, and correctly mapping the features to the local frame of the original input; the steps are similar to those of 2D-affine embeddings [26,27], but ours is in three dimensions.

### 2.1. Point-Cloud and Surface Normals

For a given intensity and depth RGB-D input pair, $\left(I,D\right)$, which are registered in a 1:1 spatial relationship, we initially minimize noisy depth effects by applying a bilateral filter [42]. Then, we compute the point-cloud and surface normals, as both are frequently utilized throughout the remainder of the manuscript.

The point-cloud is given by $C\left(p\right)=D\left(p\right)K^{-1}{\left[\begin{array}{cc} p^{⊺} & 1\; \end{array}\right]}^{⊺}$, where K is the camera-intrinsic parameters matrix, and p is a pixel. The unit surface normals, $N\left(p\right)=\frac{\nabla C\left(p\right)}{{∥\nabla C\left(p\right)∥}_{2}}$, are given by the normalized gradient of the point-cloud. Since the point-cloud is parameterized by the pixel curvilinear coordinates, $p\overset{\mathrm{def}}{=}\left(u,v\right)$ denoting the horizontal and vertical directions respectively, its gradient is given by and further approximate with [43] $\nabla C\left(p\right)=\frac{\partial C\left(p\right)}{\partial u}\times \frac{\partial C\left(p\right)}{\partial v}≊{\left[\begin{array}{ccc} \frac{\partial D\left(p\right)}{\partial u} & \frac{\partial D\left(p\right)}{\partial v} & -1 \end{array}\right]}^{⊺}$. It is worth noting that the normal direction is reversed to allow a more natural, out-of-surface orientation.

### 2.2. Enumerating and Labeling Smooth Surfaces

This section describes the process for constructing an annotation map, L, to uniquely identify *k*-smooth surfaces in the RGB-D input image. Our approach involves clustering the scene surface normals using nonparametric spherical *k*-means and then identifying the smooth surfaces by projecting the clusters back to their corresponding pixel space. We perform the latter by swapping the *domain* and *range* of the surface normals map, thus constructs an inverse map to the image space pixels, n→{p}.

Banerjee et al. proposed a spherical *k*-means [41] method for clustering on a unit hypersphere. The concept of spherical *k*-means is very similar to that of the standard *k*-means [44], except that the centroids are normalized after each expectation–maximization step. With the input being the scene surface tangent vectors (i.e., the surface normals computed in Section 2.1), we obtain clusters corresponding to the smooth surfaces of the scene. However, *k*-means is a parametric algorithm, which presents another distinct problem: determining the appropriate number of clusters, *k*.

**Figure 2:**
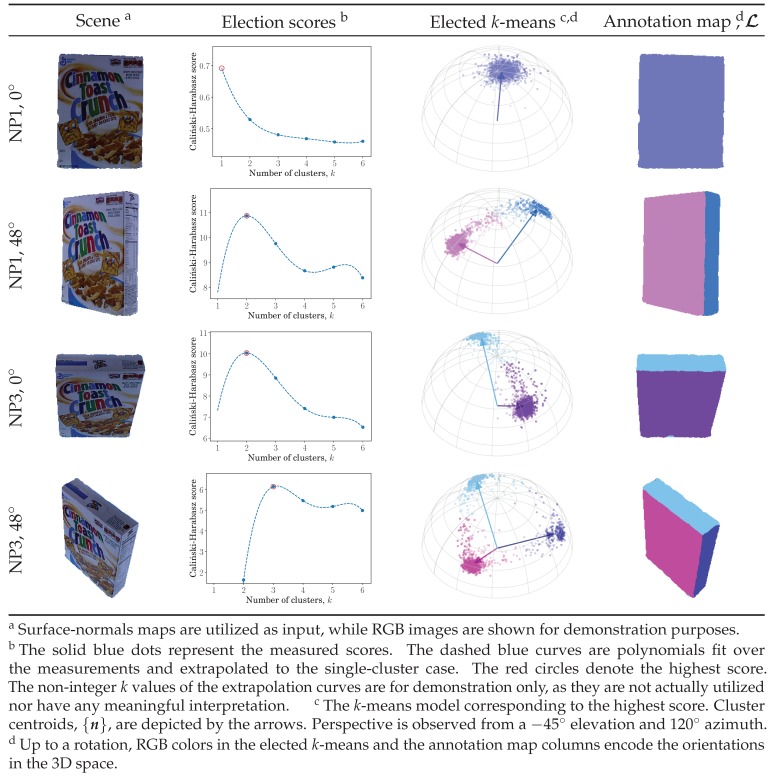
Clustering on the unit hemisphere. For each input, several spherical *k*-means models were constructed in parallel from the surface-normal map. Their Caliński–Harabasz scores were determined, where the score for the single-cluster was extrapolated. The highest-scoring *k*-means model was selected, and its clusters were back-projected to the pixel space to form the annotation map. NP1 and NP3 are two cameras fixed at 88.4° and 38.1° elevation angles, respectively.

To determine the number of smooth surfaces corresponding to *k* in the *k*-means approach, we utilize the Caliński–Harabasz score [45], which measures the within-cluster to between-cluster dispersion ratio. Different *k*-means models are constructed in parallel over a range of *k* so that the best model can be selected according to the highest Caliński–Harabasz score. Since the Caliński–Harabasz score requires at least two clusters, we form an exact-fit polynomial over the first few obtained scores and extrapolate it for the single-cluster case. Following this nonparametric approach, an appropriate spherical *k*-means clustering model is accordingly chosen. Back-projection of the chosen model’s clusters forms the annotation map, L, while its centroids, $\left\{n_{i}\right\}$, are utilized in the warping transform estimation (Section 2.3). See Figure 2 for a visualized demonstration.

### 2.3. Per-Surface Warp Transforms

For the *i*th smooth-surface, $L_{i}$, the aim is to transform the smooth surface to produce a viewpoint invariant representation, thus facilitating the computation of invariant features. In this section, the surface-warping 6D pose, $T_{i}$, is computed as a preliminary step to warp the *i*th smooth surface into a viewpoint invariant representation (Section 2.4) by aligning a smooth surface parallel to and centered at a reference local plane (e.g., the virtual image plane). The exact same process is performed in parallel for all surfaces.

**Figure 3:**
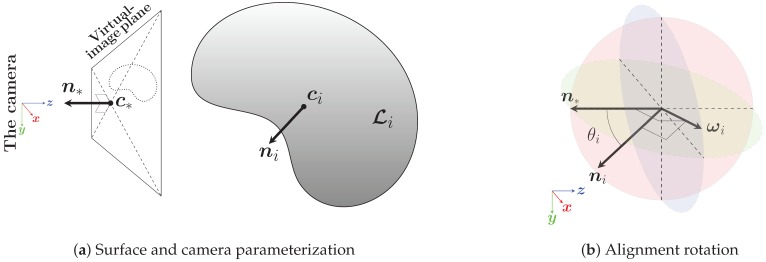
A smooth surface, $L_{i}$, is parameterized by its mass and orientation centroids, $c_{i}$ and $n_{i}$, respectively. The surface is aligned to and centered on a the virtual image plane (similarly, parameterized by $c_{*}$ and $n_{*}$) by performing: (1) a rotation of $\theta_{i}=\arccos\left(n_{*}^{⊺}n_{i}\right)$ degrees around the $\omega_{i}=n_{*}\times n_{i}$ axis and (2) a translation of $t_{i}=c_{*}-\exp\left(\theta_{i}{\left[\omega_{i}\right]}_{\times }\right)c_{i}$.

First, the smooth surface is represented by its mass and orientation centroids (Figure 3). Then, a standard plane–plane alignment method is applied. The mass centroid $c_{i}$ is expressed as the average of the surface cloud points, $C_{i}$, whereas the orientation centroid $n_{i}$ is given by the *i*th centroid of the spherical *k*-means estimation (Section 2.2).

Since $T_{i}\overset{\mathrm{def}}{=}\left(\begin{array}{cc} R_{i} & t_{i} \end{array}\right)$ is a composition of both a rotation matrix and a translation vector, they are derived separately. The rotation required to align the smooth surface to the virtual image plane is expressed first in the axis–angle ${\left(\omega ,\theta \right)}_{i}$ representation, and it is then expanded into a rotation matrix. The unit rotation axis is given by $\omega_{i}=n_{*}\times n_{i}$, while the rotation angle is $\theta_{i}=\arccos\left(n_{*}^{⊺}n_{i}\right)$, where $n_{*}$ is the orientation of the virtual image plane. The rotation matrix is an exponential map of the rotation axis–angle formalism, $R_{i}=\exp\left(\theta_{i}{\left[\omega_{i}\right]}_{\times }\right)$, which is efficiently expanded using Rodrigues’ rotation formula ([46], p. 28), $R_{i}=I+{\left[\omega_{i}\right]}_{\times }\sin\left(\theta_{i}\right)+{\left[\omega_{i}\right]}_{\times }^{2}\;\left(1\;-\cos\left(\theta_{i}\right)\right)$. The translation vector, $t_{i}=c_{*}-R_{i}c_{i}$, is formulated to center the smooth surface at the center point of virtual image plane, $c_{*}$. If a scale change is desired, the smooth surface can instead be centered at a non-unit z-vector.

### 2.4. Smooth-Surface Warping

In the previous section, the warp transform, $T_{i}$, needed to bring the smooth surface into a viewpoint invariant representation was computed. In order to apply that transform to the surface depth and intensity maps, this section investigates two major warping methods (Section 2.4.1 and Section 2.4.2). However, each of which has its own limitations, including outlier extrapolation, non-uniform pixel grids, and invalid depth mapping. Thus, we propose a hybrid method (Section 2.4.3) between the two to overcome their individual limitations. The proposed warping method is performed in parallel on all surfaces.

#### 2.4.1. Rigid-Body Warping

The rigid-body transform can easily warp the depth and intensity maps to the desired orientation and position, as expressed in the following equations:

(1) $$\begin{array}{cc} D_{i}^{\left[T\right]}\left(p^{\left[T\right]}\right){\left[\begin{array}{cc} {\left(p^{\left[T\right]}\right)}^{⊺} & 1\; \end{array}\right]}^{⊺} & =PT_{i}{\left[\begin{array}{cc} C_{i}{\left(p\right)}^{⊺} & 1\; \end{array}\right]}^{⊺},\\ I_{i}^{\left[T\right]}\left(p^{\left[T\right]}\right) & =I_{i}\left(p\right), \end{array}$$

where $D_{i}^{\left[T\right]}\left(p^{\left[T\right]}\right),p^{\left[T\right]}$ are the new depth and pixel position after applying the rigid-body transform, respectively, and $P\overset{\mathrm{def}}{=}K\left(\begin{array}{cc} I_{3} & 0_{3} \end{array}\right)$ is the camera projection matrix.

**Figure 4:**
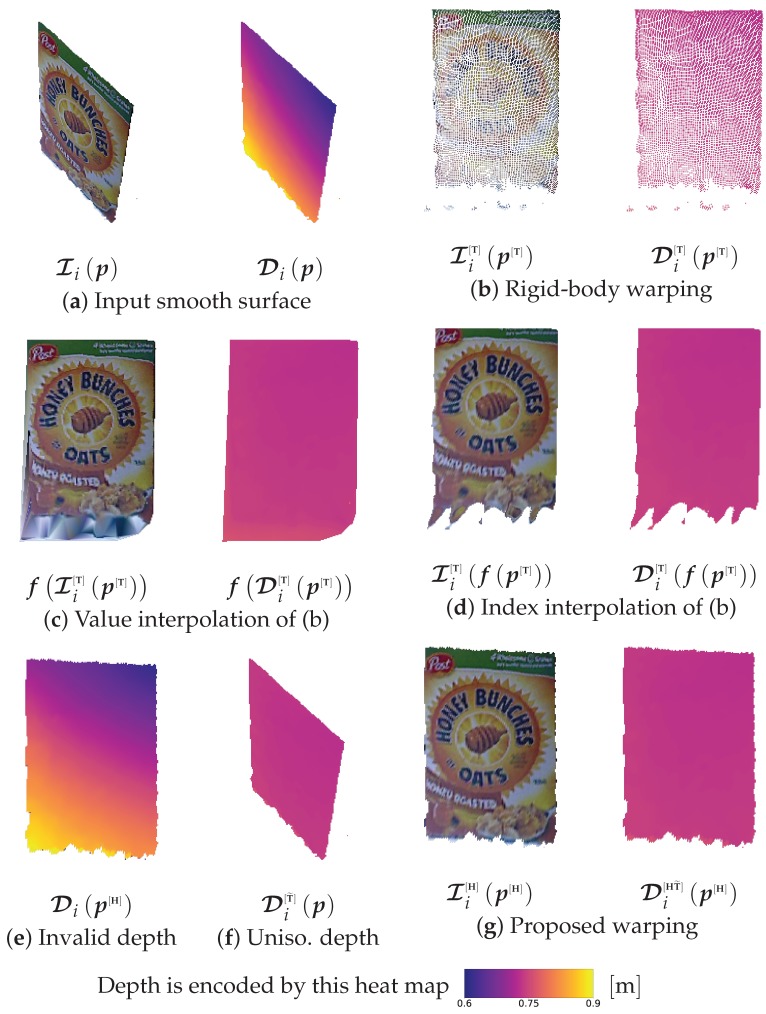
RGB-D warping techniques and the proposed improvement. (**a**) a smooth-surface tuple, ${\left(I,D\right)}_{i}$, that is not parallel to the virtual image plane, which can be (**b**) rigidly warped parallel to the virtual image plane; however, this produces many missing-value pixels, as well as some outliers (Section 2.4.1). Common approaches to fill the missing pixels include (**c**) value interpolation or (**d**) index interpolation, where $f\left(\;\cdot \;\right)$ denotes the interpolation operator. Although interpolation can fill intra-point spaces, it also extrapolates spaces between the smooth surface and the boundary outliers, thereby creating artifacts. However, because the warped points represent a smooth surface, homography is investigated; (**e**) planar homography results in invalid depth warping. Therefore, a hybrid method is proposed, in which (**f**) an unisomorphic rigid warping to the depth map is computed, followed by a homography transformation; resulting in (**g**) the proposed hybrid rigid-homography warping (Section 2.4.3).

After rigid-body warping, the warped surface may have several defects which affect the quality of the resulting image (Figure 4b). These defects include missing-value pixels due to surface out-of-plane rotation, non-uniform pixel grids from noisy depth measurements, and boundary outliers due to inaccurate surface labeling. Although linear-value interpolation or even cubic spline index interpolation can overcome missing-value issues to some extent, these methods also extrapolate to boundary outliers creating artifacts (Figure 4c,d). Fortunately, planar homography warping circumvents the non-uniform pixel grid and boundary outlier issues, as discussed next.

#### 2.4.2. Planar Homography Warping

The planar homography transform, in terms of rotation and translation, is expressed as ([47], p. 327):

(2) $$H_{i}=K\left(R_{i}-\frac{t_{i}n_{i}^{⊺}}{|c_{i}^{⊺}n_{i}|}\right)K^{-1},$$

where ${\left(R,t,n,c\right)}_{i}$ are as in Section 2.3. After this transform, the warped pixel position $p^{\left[H\right]}$ is obtained ([47], p. 33), and the RGB-D frame is morphed accordingly:

(3) $$\begin{array}{cc} s{\left[\begin{array}{cc} {\left(p^{\left[H\right]}\right)}^{⊺} & 1\; \end{array}\right]}^{⊺} & =H_{i}{\left[\begin{array}{cc} p^{⊺} & 1\; \end{array}\right]}^{⊺},\\ D_{i}^{\left[H\right]}\left(p^{\left[H\right]}\right) & =D_{i}\left(p\right),\\ I_{i}^{\left[H\right]}\left(p^{\left[H\right]}\right) & =I_{i}\left(p\right), \end{array}$$

where *s* is a scale factor introduced by unnormalized homography transforms, and $p^{\left[H\right]}$ denotes the newly obtained pixel coordinates after the homography warping. Despite homography warping being more straightforward compared with rigid-body warping (Section 2.4.1), it fails to properly map the depth values $D_{i}^{\left[H\right]}\left(p^{\left[H\right]}\right)$ because it operates in two dimensions, and therefore it is not directly feasible to obtain a warped depth map in the aligned plane (Figure 4e). Therefore, a rigid warping of the depth map is indispensable. On the other hand, boundary outliers, such as those at the bottom of Figure 4b, will be kept adjacent to the homographically morphed surface, with no intra-points between them. Thus, unlike in rigid warping extrapolation (Figure 4c,d), interpolation of planar homography warping will not locate any outlier intra-points to extrapolate. Similarly, homography implies a plate constraint on the points; thus, a uniform pixel grid is achieved despite depth noise. In light of these strengths and weaknesses of the individual approaches, a hybrid approach that takes advantage of both methods is proposed in Section 2.4.3.

#### 2.4.3. Hybrid Rigid–Homography Warping

Knowing that rigid warping does not affect the distances or angles between points, the surface can be approximated using homography after a rigid transform. Accordingly, we propose a hybrid rigid–homography method to achieve accurate and high-quality surface warping, which is summarized by the following:

(4) $$D_{i}^{\left[H\tilde{T}\right]}\left(p^{\left[H\right]}\right)=D_{i}^{\left[\tilde{T}\right]}\left(p\right)=D_{i}^{\left[T\right]}\left(p^{\left[T\right]}\right),$$

where $D_{i}^{\left[\tilde{T}\right]}\left(p\right)$ is an intermediate depth map with rigidly and unisomorphically transformed depth values. Its pixel positions are unchanged (Figure 4f), so it does not suffer from missing-value pixels as occurs with rigid-body warping (Section 2.4.1). Then, the homography transform (Section 2.4.2) is applied to the unisomorphic map, thereby properly warping the RGB-D frame (Figure 4g), with a uniform pixel grid even in presence of some noise, since homography imposes a flat-surface constraint.

**Figure 5:**
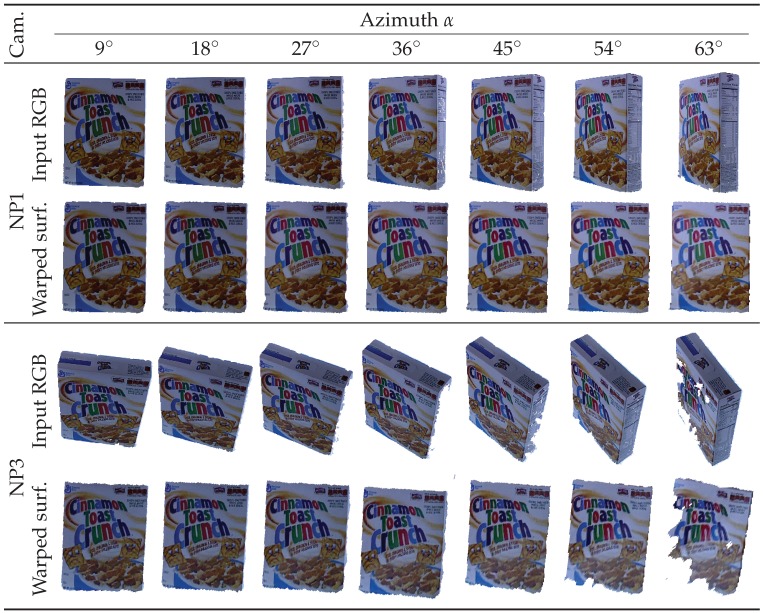
Warped smooth surfaces representing the frontal surface of a given sample. Despite various elevation and azimuth angles of the RGB-D input, smooth surfaces remain aligned to the virtual image plane. NP1 and NP3 are two cameras fixed at 88.4° and 38.1° elevation angles, respectively. Although depth maps are also warped, only RGB images of the frontal surface are shown here.

Subsequently, robust viewpoint invariant smooth surfaces are obtained for different viewpoints of the same surface (Figure 5). Although only $I_{i}^{\left[H\right]}$ is required for feature detection (Section 2.5), $D_{i}^{\left[H\tilde{T}\right]}$ will be utilized later for keypoint remapping (Section 2.6). Finally, it is worth noting that surfaces with large out-of-plane rotations may not have ideal depth maps (e.g., the camera denoted by NP3 with α = 63° shown in Figure 5 has a large missing-depth region). In that case, we interpolate the missing depth in small regions and forgo the computation of keypoints in largely missing-depth regions.

### 2.5. Feature Detection and Description

At this stage, the smooth surfaces are in a viewpoint invariant representation; they are independent from the viewpoint. Therefore, fully affine keypoints and feature description vectors can, in parallel, be extracted and computed from the smooth surfaces by applying standard detection and description processes for each smooth surface, $I_{i}^{\left[H\right]}$. Accordingly, virtually any detector and descriptor pair of choice can be utilized without regard to its invariance formula(s) or implementation.

Detectors of local image features express keypoints in image space parameters, which are passed to the feature descriptors to compute their corresponding description vectors. The feature description of each detected keypoint, despite being in arbitrary dimensions (implementation dependent), is directly utilized in correspondence estimation (Section 3.1) without requiring any adaptations. However, keypoints from different surfaces have different transforms despite representing the same scene, so combining them remains an issue, which is tackled in the next section.

### 2.6. Mapping Keypoints to Their Original Local Frame

**Figure 6:**
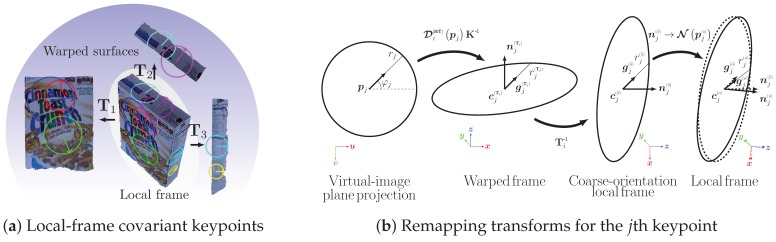
(**a**) keypoints detected on the warped surfaces are remapped back to the original input local frame to eliminate their per-surface parameterization. The resulting elliptical keypoints not only resemble those of affine region detectors but also vary covariantly with the object geometry and are invariant to viewpoint changes; (**b**) each keypoint is remapped from the virtual image plane of the smooth surface viewpoint invariant representation to the local frame of the original input by applying several transforms. The keypoint is first unprojected to the 3D space (Section 2.6.1), then its basis is changed to the local frame (Section 2.6.2). Finally, its orientation is corrected (Section 2.6.3). Refer to Section 2.6 for annotation and technical details.

This step is intended to simplify computations by eliminating the dependency of each keypoint on its per-frame transform, $T_{i}$ (Section 2.3), based on the fact they all belonged to the same scene before mapping (Figure 6a). Putting together all keypoints in the local frame of the RGB-D input prepares them for correspondence estimation with other images and also results in elliptical affine region neighborhoods that are compatible with affine region detectors [48].

Each *j*th detected keypoint ${\left(p,\varphi ,r\right)}_{j}$ is initially expressed using image space parameters ${\left(\;\cdot \;\right)}_{j}$, where $p_{j}\in \;R^{2}$ is the keypoint center, $\varphi_{j}\in \;R$ is the dominant gradient orientation [19], and $r_{j}\in \;N$ is the neighborhood radius in pixels. To map the keypoints from the image frame ${\left(\;\cdot \;\right)}_{j}$, to the local frame ${\left(\;\cdot \;\right)}_{j}^{\left(o\right)}$, two intermediate parameterizations are utilized: the warped-frame ${\left(\;\cdot \;\right)}_{j}^{\left(T_{i}\right)}$, and the coarse orientation of keypoints in the local frame ${\left(\;\cdot \;\right)}_{j}^{\left(\tilde{o}\right)}$ (see Figure 6b). We describe these three steps in the following sections. The exact same process is performed in parallel for all keypoints.

#### 2.6.1. Image to Warped Frame Transformation

This section re-parameterizes each detected 2D keypoint ${\left(p,\varphi ,r\right)}_{j}$ on the warped surfaces into 3D-space parameters ${\left(c,n,g,r\right)}_{j}^{\left(T_{i}\right)}$ of the corresponding *i*th warped frame, where $T_{i}$ is the per-surface warp transform (Section 2.3), $c_{j}^{\left(T_{i}\right)}\in \;R^{3}$ is the keypoint center, $n_{j}^{\left(T_{i}\right)}$ is the plane tangent orientation, $g_{j}^{\left(T_{i}\right)}\in \;R^{3}$ is the unit gradient orientation, and $r_{j}^{\left(T_{i}\right)}\in \;R^{}$ is the radius. Without loss of generality, at this stage, we assume that keypoints are parallel to the virtual image plane, $n_{j}^{\left(T_{i}\right)}=n_{*}$ (Section 2.3), to ensure their orthogonality with the gradient orientation, $g_{j}^{\left(T_{i}\right)}$. We will compensate for this orientation assumption by Equation (7) in a subsequent section. The rest of the parameters, ${\left(c,g,r\right)}_{j}^{\left(T_{i}\right)}$, are given by:

(5) $$\begin{array}{cc} c_{j}^{\left(T_{i}\right)} & =D_{i}^{\left[H\tilde{T}\right]}\left(p_{j}\right)K^{-1}{\left[\begin{array}{cc} p_{j}^{⊺} & 1\; \end{array}\right]}^{⊺},\\ r_{j}^{\left(T_{i}\right)}g_{j}^{\left(T_{i}\right)} & =D_{i}^{\left[H\tilde{T}\right]}\left(p_{j}\right)K^{-1}T_{j}{\left[\begin{array}{cc} p_{j}^{⊺} & 1\; \end{array}\right]}^{⊺}-c_{j}^{\left(T_{i}\right)}, \end{array}$$

where $r_{j}^{\left(T_{i}\right)}$ and $g_{j}^{\left(T_{i}\right)}$ are separable due to ${∥g_{j}^{\left(T_{i}\right)}∥}_{2}=1$, and $T_{j}=\left(\begin{array}{cc} I_{3\;\times 2} & {\left[\begin{array}{ccc} r_{j}\cos\;\varphi_{j} & r_{j}\sin\;\varphi_{j} & 1\; \end{array}\right]}^{⊺} \end{array}\right)\in \;{\mathrm{SE}}_{2}$ is an image transform that shifts $p_{j}$ by $r_{j}$ in the $\varphi_{j}$ direction. Note that $p_{j}$ is a tuple of real numbers, in which the depth value $D_{i}^{\left[H\tilde{T}\right]}\left(p_{j}\right)$ is interpolated over the neighborhood of $p_{j}$.

#### 2.6.2. Warped to Coarse-Orientation Local Frame Transformation

By applying the inverse of the smooth-surface transform, $T_{i}\overset{\mathrm{def}}{=}\left(\begin{array}{cc} R_{i} & t_{i} \end{array}\right)$, to the warped frame parameters ${\left(c,n,g,r\right)}_{j}^{\left(T_{i}\right)}$, the coarse-orientation local parameters ${\left(c,n,g,r\right)}_{j}^{\left(\tilde{o}\right)}$ are obtained, where radius, $r_{j}^{\left(\tilde{o}\right)}=r_{j}^{\left(T_{i}\right)}$, remains unchanged, and ${\left(c,n,g\right)}_{j}^{\left(\tilde{o}\right)}$ are given by:

(6) $$\begin{array}{cc} {\left[\begin{array}{cc} {\left(c_{j}^{\left(\tilde{o}\right)}\right)}^{⊺} & 1\; \end{array}\right]}^{⊺} & =T_{i}^{-1}{\left[\begin{array}{cc} {\left(c_{j}^{\left(T_{i}\right)}\right)}^{⊺} & 1\; \end{array}\right]}^{⊺},\\ \left[\begin{array}{cc} n_{j}^{\left(\tilde{o}\right)} & g_{j}^{\left(\tilde{o}\right)} \end{array}\right] & =R_{i}^{⊺}\left[\begin{array}{cc} n_{j}^{\left(T_{i}\right)} & g_{j}^{\left(T_{i}\right)} \end{array}\right]. \end{array}$$

#### 2.6.3. Coarse-to-Fine Local Frame Transformation

Among the local frame parameters ${\left(c,n,g,r\right)}_{j}^{\left(o\right)}$, center and radius, ${\left(c,r\right)}_{j}^{\left(\tilde{o}\right)}={\left(c,r\right)}_{j}^{\left(o\right)}$ are exactly the same as the corresponding coarse-orientation local parameters, and only tangent and gradient orientations ${\left(n,g\right)}_{j}^{\left(\tilde{o}\right)}$ needs to be corrected due to the orientation assumption made earlier. It is worth noting that (1) any rigid transform preserves orthogonality, (2) by the construction of Section 2.6.1, ${\left(n_{j}^{\left(T_{i}\right)}\right)}^{⊺}g_{j}^{\left(T_{i}\right)}=0$; therefore, the unit vectors $\left(n_{j}^{\left(T_{i}\right)},g_{j}^{\left(T_{i}\right)},n_{j}^{\left(T_{i}\right)}\times g_{j}^{\left(T_{i}\right)}\right)$ form an orthogonal basis. Accordingly, the flat-plane assumption is compensated for by replacing $n_{j}^{\left(\tilde{o}\right)}$ with the correct surface normal (as computed in Section 2.1) and thereupon correcting $g_{j}^{\left(\tilde{o}\right)}$. Consequently:

(7) $$\begin{array}{cc} n_{j}^{\left(o\right)} & =N\left(p_{j}^{\left(o\right)}\right),\\ g_{j}^{\left(o\right)} & =n_{j}^{\left(\tilde{o}\right)}\times \left(g_{j}^{\left(\tilde{o}\right)}\times n_{j}^{\left(o\right)}\right), \end{array}$$

where $p_{j}^{\left(o\right)}\in \;R^{2}$ is the projection of $c_{j}^{\left(o\right)}$ on the virtual image plane, i.e., $D\left(p_{j}^{\left(o\right)}\right){\left[\begin{array}{cc} {\left(p_{j}^{\left(o\right)}\right)}^{⊺} & 1\; \end{array}\right]}^{⊺}=Kc_{j}^{\left(o\right)}$, by which the surface-normal map is interpolated.

Thus far, we achieved invariant detection and description, in which the embedding returns the feature description vector (depending on the descriptor’s own implementation). Furthermore, it returns a 3D keypoint tuple ${\left(c,n,g,r\right)}_{j}^{\left(o\right)}$ for each detected and described 2D keypoint ${\left(p,\varphi ,r\right)}_{j}$, given the smooth-surface transform $T_{i}$ (Section 2.3) and its depth map $D_{i}^{\left[H\tilde{T}\right]}$ (Section 2.4.3). The proposed keypoint parametric representation is useful for enriching additional tasks, such as 3D matching (Section 3) and elliptical neighborhood projection (Figure 6a), commonly used by affine region detectors [48].

## 3. An Application: 6D Pose Estimation

**Figure 7:**
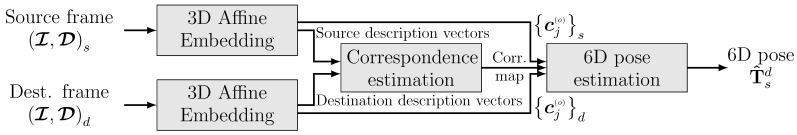
6D pose estimation is one high-level application, among many, that can utilize the proposed embedding as a building block. First, feature description vectors are utilized to compute the correspondence (Section 3.1). The 6D pose is then computed from the correspondence map (corr. map) and keypoint centers via geometric verification (Section 3.2).

We chose the challenging 6D pose estimation problem as a high-level algorithm in which the proposed embedding acts as a building block. See Figure 7 for an abstraction. Given two tuples of source and destination RGB-D images, ${\left(I,D\right)}_{s}$ and ${\left(I,D\right)}_{d}$, the aim is to find the relative position and orientation, i.e., the 6D pose ${\hat{T}}_{s}^{d}$ ([46], pp. 36–37), that properly registers these partial, so-called 2.5D, scenes together. The 6D pose correspondences, ${\hat{T}}_{s}^{d}$, between the source and destination feature vectors are established using the standard *k*-NN (*k*-Nearest Neighbor) algorithm [49]. A 3D space geometric verification method [50] is employed with a RANSAC (Random Sample Consensus) [51] variant called Optimal RANSAC [52] in order to achieve robustness to correspondence outliers between the keypoint centers. We describe this in further detail in the following subsections.

### 3.1. Correspondence Estimation

Given two sets of description vectors—namely, the source and destination—and using the *k*-NN algorithm [49], a tree-based index is built from one set and then all of the members in the other set are queried for the *k*-NN in the index. We computed the *k*-NN, where k=1, from one set to another and vice versa, and then limited correspondence to mutual *k*-NNs. The correspondence can be filtered further by thresholding the ratio of first-to-second nearest-neighbor distances [19]. After obtaining the correspondence map between the two sets, the keypoints from both sets are grouped into corresponding pairs in order to estimate the 6D pose (Section 3.2).

### 3.2. Geometric Verification and 6D Pose Estimation

Utilizing the center point, and the normal and gradient orientations of the corresponding keypoints, $\left\{{\left(c,n,g\right)}_{j}^{\left(o\right)}\right\}$ (Section 2.6.3), the 6D pose can be estimated from only a single correspondence [1]. However, not all detectors produce sufficiently large keypoints; the transformation robustness is highly dependent upon the keypoint gradient, and a large enough neighborhood is essential for stable estimation. Furthermore, the standalone local image features, our points of comparison, do not provide normal and gradient orientations that are viewpoint invariant. Therefore, we sacrifice the utilization of normal and gradient orientations and only utilize the keypoint centers, $\left\{c_{j}^{\left(o\right)}\right\}$, in order to enable a fair comparison. For the aforementioned reason, the point-cloud (Section 2.1) is computed for standalone cases to interpolate their 3D keypoint centers.

Verification was performed by forming a rigidity constraint out of two corresponding sets of 3D keypoint centers, each containing three points at minimum. A SVD (Singular Value Decomposition)-based method [50] is employed to compute the 6D pose ${\hat{T}}_{s}^{d}$, within the Optimal RANSAC iterations, as it acts as the fitting model for the hypothetical inliers.

## 4. Experimental Setup

In Section 2, we proposed an embedding to improve the viewpoint invariance of virtually any local image detector/descriptor without requiring any adaptations to their interfaces or internal invariance formulas. This section is dedicated to the experimental setup to demonstrate the proposed method’s effectiveness, generality, and robustness by performing two types of experiments: performance comparison and sensitivity analysis.

The performance in all experiments was assessed using viewpoint invariance scores (Section 4.3). Viewpoint invariance measures the stability against viewpoint changes by counting the number of out-of-plane samples for which a correct estimation of the relative pose is achieved. The samples consists of several pairs of images, a source and a destination at each iteration, taken from a wide interval of different relative viewpoints. Additionally, because there are randomized factors that stem from *k*-means initialization (proposed embedding only) and Optimal RANSAC [52] (standalone and embedded), each experiment, for both standalone and embedded approaches, was performed in triplicate, and the mean and standard deviation of the performance metric are reported.

The proposed embedding was implemented in Python™ [53]. Our implementation widely depended on the NumPy [54], OpenCV [55], Scikit-learn [56], and SciPy [57] packages for linear algebra, image processing, modeling, and signal processing, respectively. Furthermore, the Matplotlib [58] and MayaVi [59] packages were used for 2D and 3D graphics, respectively.

### 4.1. Performance Comparison Experiments

**Figure 8:**
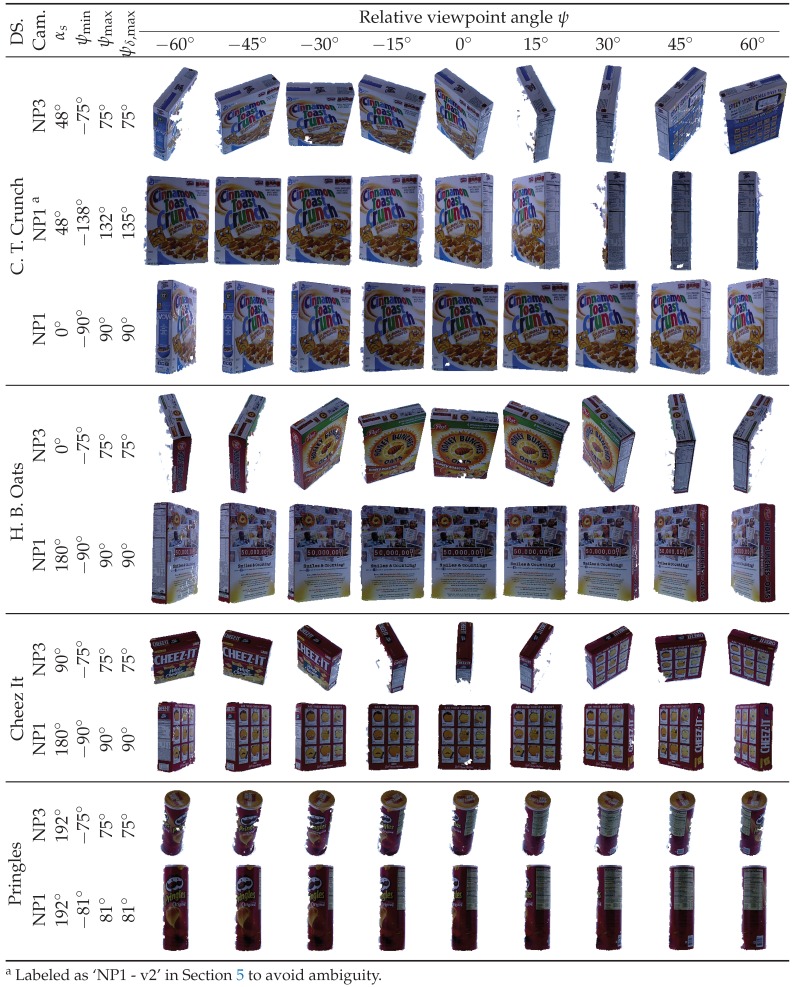
RGB images of the evaluation datasets (DS.) from different cameras (Cam.) sampled along different relative viewpoint angles, ψ. The source image is fixed to $\alpha_{s}$ (equivalent to ψ = 0°), while the destination image is taken from the interval [$\psi_{\min}$, $\psi_{\max}$] at every 3° step. Based on the source frame and camera elevation, each dataset limits the viewpoint invariance score, $\psi_{\delta }$, to a specific maximum, $\psi_{\delta ,\max}$. Only sample images are shown.

We first performed a brief comparison in an ideal synthetic setup in order to show the extended level, in terms of the viewpoint invariance range, of the proposed embedding compared with the well-known SURF detector and SIFT descriptor. The synthetic dataset consisted of a cuboid object representing a cereal box, where its rendered RGB-D frames resembles both texture and viewpoints of the real-world ‘C. T. Crunch’ object observed from the NP1 camera (Figure 8). After the synthetic experiment, several state-of-the-art feature detectors and descriptors were embedded within the proposed method to establish its generality. At the same time, to demonstrate the effectiveness of the proposed embedding, each embedded detector/descriptor was compared with its standalone version. A list of the detectors and descriptors we studied, at the standalone and embedded levels, is provided in Table 2. Because it is impractical to evaluate all intermixed combinations, we tested the performance of all detectors using SIFT as the representative descriptor and we tested all descriptors using SURF as the representative detector. We chose these since both SURF and SIFT are common feature detectors/descriptors and because SURF has reasonably large keypoints.

To ensure realistic results with different settings, this comparison was performed using real-world objects with planar and curved surfaces, various texture patterns, and different illumination conditions. Four objects were chosen, each of which has a different texture, color intensities, and contrast. The first three have box-like polygonal shapes to represent planar surfaces, and the fourth is cylindrical to represent curved surfaces. All objects were rotated in wide-azimuth rotations and were observed by cameras at two different elevations, from which viewpoint invariance was evaluated. At the same time, illumination conditions change on the objects’ sides while they rotate. We also investigated the effects of the source frame selection by repeating an experiment with a different source frame. The 960 RGB-D frames representing the four objects (Figure 8) are instances of the BigBIRD datasets [60] and were captured using intrinsically and extrinsically calibrated RGB-D cameras fixed at different elevation angles. Over the entire 360° rotation window for the motorized turntable below the target object, images were captured for each 3° azimuth step by all cameras. Furthermore, the datasets provide ground-truth data which define the object region in the image, i.e., binary annotation maps, $L_{k}\left(p\right)\in \;\left\{0,1\;\right\}$. Refer to Reference [60] for further details.

The datasets utilized in our evaluation are ‘C. T. Crunch’, ‘H. B. Oats’, ‘Cheez It’, and ‘Pringles’, which represent some polygonal and cylindrical objects. The cameras used for evaluation are the NP1 and NP3 cameras, which were fixed at 88.4° and 38.1° elevation angles, respectively. Dataset intrinsic and extrinsic parameters were utilized for 1:1 depth-to-color registration. These parameters were also used with the turntable azimuth angle to compute the ground-truth of relative pose transform $T_{s}^{d}\overset{\mathrm{def}}{=}\left(\begin{array}{cc} R_{s}^{d} & t_{s}^{d} \end{array}\right)$ between any two RGB-D pairs, ${\left(I,D\right)}_{s}$ and ${\left(I,D\right)}_{d}$. The background was masked using the dataset annotation maps, $L_{k}$.

Sample dataset frames, along with their relative viewpoint angles, are shown in Figure 8. Note that the relative viewpoint angles are well beyond ±60°, as denoted in Figure 8 by the [$\psi_{\min}$, $\psi_{\max}$] interval. It is also worth noting that the second and third rows in Figure 8, which differ in source frame, are intended to investigate the effects of the source frame selection. Furthermore, note the realistic setup, in which the missing-depth instances and different illumination effects are quite apparent.

### 4.2. Sensitivity Analysis Experiments

A depth-noise sensitivity analysis was performed during viewpoint changes in the following manner. A synthetically ideal RGB-D frame was matched against several other frames, each of which has a noisy depth and a different viewpoint angle. In this experiment, the response to numerous SNRs (Signal-to-Noise Ratios) was studied, with SURF and SIFT as the feature detector and descriptor, respectively. To obtain a noisy depth map with a specific SNR, the standard definition was utilized, $\mathrm{SNR}=20\;\log_{10}\left(\frac{\mu }{\sigma }\right)$, where μ is the signal mean and σ is the noise deviation. More specifically, a synthetic ideal depth map of the scene geometry was elementwise multiplied by samples drawn at random from a normal distribution, $N\left(\mu ,\sigma^{2}\;\right)$, with mean μ=1 and variance σ2=10−SNR10. A sufficiently large range of SNRs was considered, namely, from the range 20 dB–65 dB in steps of 5 dB.

### 4.3. Performance Metric

Although local image features are usually evaluated in terms of detection repeatability [48] and description distinctiveness [61], these indices do not seem to be very accurate indicators of the performance of a local image feature when it is utilized in other high-level algorithms, which is often the case. That is, in high-level problems, there is no guarantee that a pair comprising a highly repeatable detector and a highly distinct descriptor can perform better than other combinations of detectors and descriptors. Admittedly, repeatability is measured as the sampling window intersection-to-union ratio of corresponding keypoints, which is misleading for applications that depend only on the center point, such as correspondence estimation. For instance, two concentric keypoints with different radii are deemed to have a low repeatability despite being a perfect correspondence pair. Similarly, two elliptical keypoints with high aspect ratios and with their centers located along each other’s major axis would be designated with moderately high repeatability, even if their centers are far apart. Therefore, the performance of each detector/descriptor pair was evaluated at the application level using a high-level performance indicator.

Accordingly, we opt to utilize the stability against different viewpoint changes as a performance metric. Briefly, it measures the accumulative-interval length along the axis of a relative viewpoint angle, for which the alignment error is tolerated. In this section, as a preliminary step to introduce the performance metric of viewpoint invariance, we need to first define the relative viewpoint angle, the transform estimation error, and the alignment error.

The relative viewpoint angle, ψ, is defined as the source–destination out-of-plane rotation angle:

(8) $$\psi =\arccos\left(n_{*}^{⊺}R_{s}^{d}n_{*}\right),$$

where $n_{*}$ is the virtual image plane orientation (Section 2.3). Note that ψ is also assigned the azimuth sign to indicate the rotation direction.

The transform estimation error, $T_{e}\in \;{\mathrm{SE}}_{3}$, measures the difference between the estimated 6D pose, ${\hat{T}}_{s}^{d}$, and that of the ground truth, $T_{s}^{d}$, while the alignment error, $\ell \left(\psi \right)\in \;R$, is the RMS (Root-Mean-Square) of the point-to-point misalignment distance resulting from $T_{e}$. These quantities are given by:

(9) $$\begin{array}{cc} T_{e}\left(\psi \right) & =T_{s}^{d}{\left(\psi \right)}^{-1}{\hat{T}}_{s}^{d}\left(\psi \right),\\ \ell \left(\psi \right) & ={\left(\frac{1\;}{n}\sum_{p}{∥T_{e}\left(\psi \right)\left[\begin{array}{c} C_{s}\left(p\right)\\ 1\; \end{array}\right]-\left[\begin{array}{c} C_{s}\left(p\right)\\ 1\; \end{array}\right]∥}_{2}^{2}\right)}^{\frac{1}{2}}, \end{array}$$

where $C_{s}$ and *n* are the point-cloud of the source frame (Section 2.1) and the number of points in it, respectively.

The performance metric of the viewpoint invariance, $\psi_{\delta }$, can be defined as the ψ-axis length where the alignment error, $\ell \left(\psi \right)$, is less than or equal to a maximally tolerated alignment error, $\ell_{\epsilon }$, up to a normalizing constant. Mathematically, let $\left\{\psi_{0},\cdots ,\psi_{n-1}\right\}$ be an *n*-element set in ascending order, containing both the dataset bounds $\left\{\psi_{\min},\psi_{\max}\right\}$ and the roots of the polynomial $\ell \left(\psi \right)=\ell_{\epsilon }$, then $\psi_{\delta }$ is given by:

(10) $$\psi_{\delta }=\frac{1}{s}\sum_{m=0}^{n-2}\left\{\begin{array}{cc} \psi_{m+1}-\psi_{m}, & \mathrm{if}\;\ell \left(\frac{\psi_{m+1}+\psi_{m}}{2\;}\right)\leq \;\ell_{\epsilon }\\ 0, & \mathrm{otherwise}, \end{array}\right.,$$

where *s* is a normalizing constant set to s=2 (assuming symmetric alignment error in both relative viewpoint directions). A detector/descriptor pair is considered viewpoint invariant if the length of its viewpoint invariance score, $\psi_{\delta }$, is greater than or equal to a given threshold, $\psi_{\Delta }$.

We set the maximally tolerated alignment error to $\ell_{\epsilon }=\sqrt{2}\;cm$ and the desired degree of viewpoint invariance to $\psi_{\Delta }$ = 60°. The maximally tolerated alignment error is constrained by factors that originate from our motivation; to perform 3D object reconstruction and grasp, the said object using a robotic manipulator, in which $\ell_{\epsilon }$ corresponds to the manipulator tolerance. Furthermore, based on images corresponding to equal cones of vision on a systematically sampled sphere, only 13 cones, and thus images, are sufficient to cover the view sphere with $\psi_{\Delta }$ = 60°, as compared with 53 cones in the case of the standalone features viewpoint limit, 25°–30°.

The performance metric, $\psi_{\delta }$, measures the viewpoint invariance by considering a wide range of viewpoint changes and quantifying their corresponding errors. It is utilized in the next section to compare the viewpoint invariance of both standalone and embedded local image features in order to show the proposed method’s effectiveness.

## 5. Results and Discussion

### 5.1. Performance Comparison Experiments

This section aims to provide an in-depth understanding of the viewpoint limitations in previous developments and the extended viewpoint range of the proposed method. First, a synthetic dataset is used. Then, the results are presented from numerous experiments that were performed using various local image features, both with and without the proposed embedding and on several real-world objects. The results are quantitatively compared, and the proposed embedding demonstrates viewpoint invariance gains for almost any local image detector/descriptor without requiring any interfacing or internal adaptations. On the basis of further qualitative results for some challenging viewpoints, the proposed embedding shows great stability in correspondence and pose estimation against viewpoint changes.

#### 5.1.1. Ideal Synthetic Data

**Figure 9:**
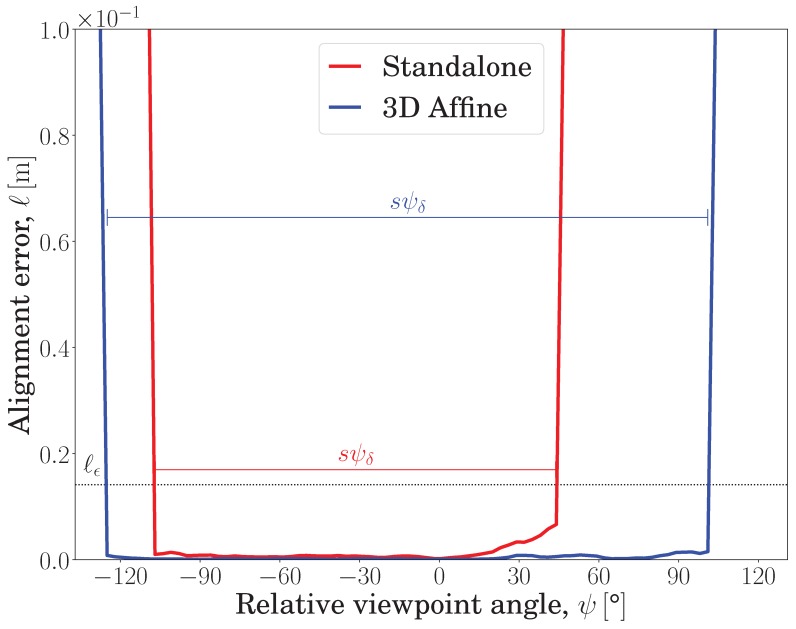
The proposed embedding, denoted by the blue curve, is compared with the standalone feature approach, represented by the red curve. The proposed embedding remained viewpoint invariant (i.e., $\ell \left(\psi \right)\leq \;\ell_{\epsilon }$) over a large interval of ψ-axis values. Thus, it outperformed the standalone intensity-based approach. The dotted horizontal line denotes the maximally tolerated alignment error, $\ell_{\epsilon }$; the longer a curve, $\ell \left(\psi \right)$, remains below this line, the larger its invariance score, $\psi_{\delta }$, where *s* is a normalizing constant. In this setting, the SURF [20] detector and the SIFT [19] descriptor were evaluated on a synthetic dataset resembling the ‘C. T. Crunch’ object observed from the NP1 camera.

After a brief comparison of both proposed and existing approaches, this section answers the question of why the proposed method behaves differently and favorably despite its dependence on the same detector and descriptor used by the standalone approach. A synthetically ideal dataset resembling the real-world ‘C. T. Crunch’ dataset (third row in Figure 8) was constructed, in which one RGB-D frame is fixed and matched against the rest of the frames using the SURF detector and SIFT descriptor for both the standalone and proposed methods. The embedded variant of the SURF/SIFT approach (*3D affine* for short) outperformed the standalone version as shown in Figure 9.

In Figure 9, the horizontal axis, ψ, represents a total of 90 matching experiments between a fixed source frame and various destination frames with different out-of-plane rotations, sampled at every 3°. The vertical axis represents the alignment error, $\ell \left(\psi \right)$, Equation (9), which is desirably kept lower than the maximally tolerated alignment error, $\ell_{\epsilon }$ (the black dotted horizontal line). The viewpoint invariance score, $\psi_{\delta }$, Equation (10), represents the length of the relative viewpoint angle range for which the alignment error curve of each approach remains below the maximally tolerated alignment error, $\ell_{\epsilon }$. Since the curve is generally symmetric around ψ = 0° (i.e., the source image corresponding to the $\alpha_{s}$ azimuth value indicated in Figure 8), a normalizing constant, s=2, is utilized to consider only half of the accumulative interval as the actual viewpoint invariance score. When using standalone features, a viewoint invariance range of $\psi_{\delta }$ = 75.89° is observed, while the proposed method achieved a higher viewpoint invariance range, $\psi_{\delta }$ = 113.53°. It is worth nothing that both the standalone and embedded features exhibit similar U-shaped curves, in which the minimum value is observed around ψ = 0°, and they approach saturation values on ψ-axis edges related to the object dimensions. Furthermore, the maximally tolerated alignment error, $\ell_{\epsilon }$, is observed to reside mostly near the elbow of the $\ell \left(\psi \right)$ curves, thereby enabling an effective and low deviation assessment of viewpoint invariance. Overall, the standalone approach was able to more-or-less keep up with our proposed method, except in the ranges of −126° to −108° and 45° to 102°. Thus, a sample matching experiment from these ranges is inspected.

**Figure 10:**
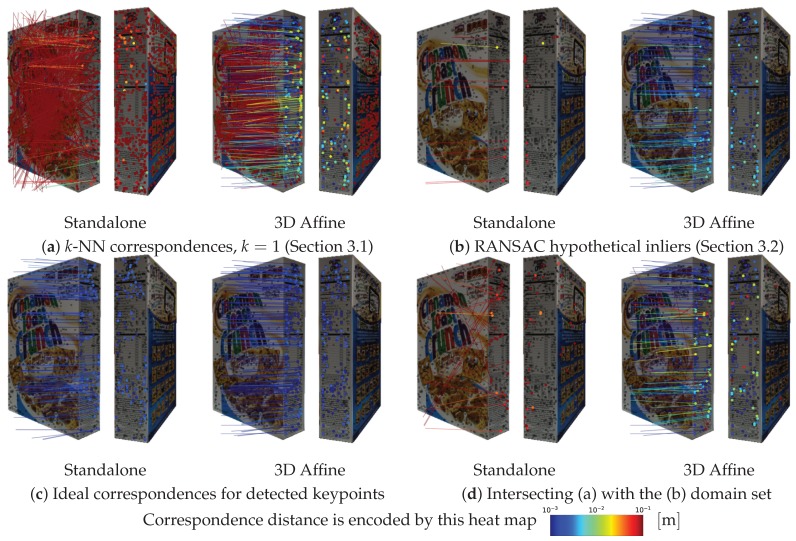
The standalone SURF [20] detector and SIFT [19] descriptor had difficulty detecting sufficient repeatable keypoints (left sides of **a**, **b**, and **c**) and computing distinct descriptions (left sides of **a**, **b**, and **d**), respectively, under relatively-large viewpoint change. The proposed embedding (3D affine) alleviated the problem by leveraging the geometric information from the depth maps to undo the viewpoint change effects (right side of **b**, refer to Section 5.1.1 for details). Correspondences are visualized as a sparse optical-flow to improve visibility.

Next, to investigate the reasons behind the weakness of the standalone approach compared with the proposed method, a poorly matched standalone case, as previously reported in Figure 9, is considered as a case study here. As a sample, ψ = 60° matching case is taken and its inlier and outlier correspondences are visualized in the blue-to-red color range depending on how far they are from the ground truth. As shown in Figure 10a, it is apparent that the standalone detector/descriptor pair has a huge outlier ratio at the correspondence stage (Section 3.1). The low inlier ratio leads to RANSAC failing to recover a proper set of inliers, as shown in Figure 10b. On the other hand, despite the proposed embedding utilizing the exact same detector and descriptor, it is able to produce better correspondences with a higher inlier ratio, thus properly filtering out the outliers at the geometric verification stage (Section 3.2).

Now, to delve further into answering why the standalone method has a low inlier ratio, the detection and description stages are investigated independently. By constructing an ideal descriptor based on the ground truth, as shown in Figure 10c, ideal correspondences can be obtained for any set of detected keypoints. The standalone detected keypoints in the corresponding region have a lower density than those of the 3D-affine methods. With an almighty descriptor and without synthesized factors (e.g., scale, illumination, blur, or contrast) apart from the viewpoint change, results suggest that the detection repeatability decreases with large viewpoint changes. On the other hand, despite using the same detector, the proposed 3D-affine embedding was able to bypass such a scenario by warping the surfaces (Section 2.4) into viewpoint-independent images, enabling a more repeatable detection from both source and destination frames.

Similarly, the descriptor can be studied independently by limiting the set of *k*-NN correspondences (Figure 10a) to those sharing the same domain set with the ideal ones (Figure 10c), as shown in Figure 10d. In this case, most of the standalone correspondences, if not all, paired with the wrong keypoint, despite the existence of an ideal corresponding keypoint in the range set. This indicates that, due to the viewpoint change, indistinct descriptions were computed for these ideally repeatable keypoints, resulting in higher matching distances than those measured with other invalid pairs. Failing to generate proper descriptions suggests that the sampling window cannot invariantly describe large skew-like neighborhood changes to handle the viewpoint changes. Even small sampling windows cannot alleviate the situation, which implies the need for the elliptical keypoints (Figure 6a). Contrarily, the proposed method renders the surfaces as viewpoint independent representations. Thus, it is bestowed with more distinctiveness (not perfect though), albeit using the same descriptor.

#### 5.1.2. Real-World Data

After the in-depth insights obtained from the previously discussed experiment, the focus is now on demonstrating the proposed method’s effectiveness and generality through extensive experiments. The discussion is grouped into three segments based on the utilized datasets, detectors, and descriptors. After that, more comprehensive results are reported, and the section is concluded with a discussion of the proposed method’s limitations. For conciseness, it is deemed sufficient to state the viewpoint invariance scores, $\psi_{\delta }$, given that most of the detector/descriptor pairs feature similar, yet noisy, tendencies to the curves in Figure 9, and their viewpoint invariance is well captured by the $\psi_{\delta }$ metric from Equation (10).

Although the viewpoint invariance depends on the detectors/descriptors invariance formula(s), it is also affected by the evaluated scene properties (e.g., texture, blur, contrast, object geometry, and the number of distinct surfaces observed on it). Furthermore, the score is affected by source frame selection, especially if repeated texture, blurry regions, or few surfaces are observed on it. To demonstrate this, a total of nine different combinations—composed of four different objects, two different elevations, and several azimuth angles (more than 120 samples each)—were utilized, as shown in Figure 8. Although the next discussion of the different datasets is limited to the representative SURF detector and SIFT descriptor, similar observations can be made for the 18 remaining detectors/descriptors pairs, as reported later in this section.

**Figure 11:**
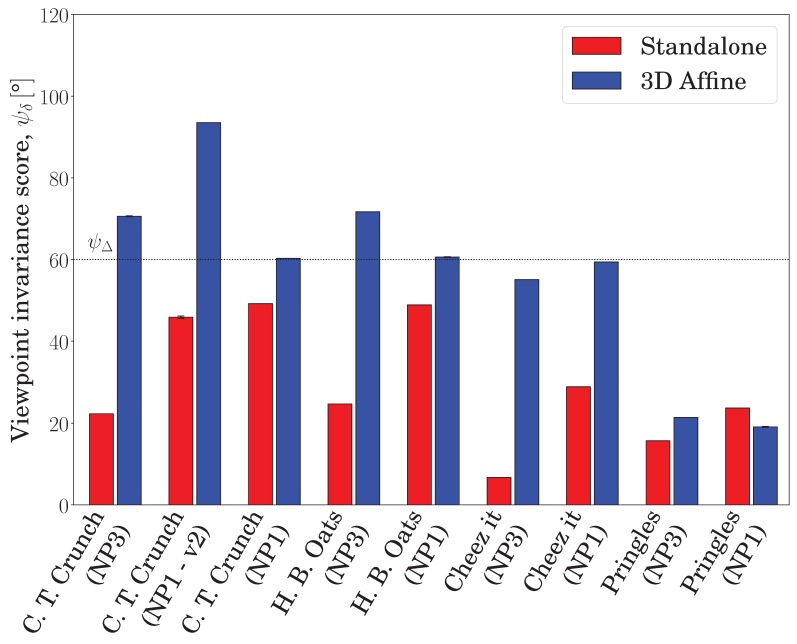
Viewpoint invariance depends on the object geometry, texture, and the number of observed distinct surfaces. The vertical axis represents the viewpoint invariance score, $\psi_{\delta }$, while the horizontal axis represents different datasets, observed from the NP1 and NP3 cameras (Figure 8). The red bars and the blue bars represent the standalone and the embedded local image features, respectively. Generally, the proposed method outperformed the standalone one for the vast majority of the datasets, where none of the standalone features approached the targeted viewpoint invariance, $\psi_{\Delta }$. The data in this figure are from experiments with the SURF [20] detector and the SIFT [19] descriptor. Refer to later parts of Section 5.1.2 for the results of other detectors/descriptors.

In Figure 11, the horizontal axis denotes different datasets and cameras, the vertical axis denotes the viewpoint invariance score, $\psi_{\delta }$, and the black dotted horizontal line denotes the goal viewpoint invariance threshold, $\psi_{\Delta }$. The standalone and embedded local image feature are denoted by the red and blue bars, respectively. The proposed embedding achieved the targeted viewpoint invariance, $\psi_{\Delta }$, for most datasets featuring polygonal objects and distinct surface discontinuities; however, the standalone feature failed to approach the target for any of the datasets. It is worth noting that the scores reported in this realistic setup are less than the equivalent scores reported in Section 5.1.1, which is a natural outcome, given that the previous experiment was in a synthetic setting. In fact, the differences in the score levels correspond to the dataset difficulty in terms of geometry, texture, blur, contrast, and the number of observed surfaces. For example, the ‘Cheez It’ dataset observed from the NP3 camera was very challenging for the standalone feature approach (only 6.7°), because it included small and repeated texture patterns; however, the proposed embedding almost succeeded (55.1°) by exploiting geometric information resident in the scene. The most challenging case for the proposed embedding involved indistinct surface discontinuity (the ‘Pringles’ dataset; see the last paragraph in this section for details).

Importantly, the more surfaces observed, the better the achieved viewpoint invariance of the embedded features, and the more deteriorated the viewpoint invariance obtained by the standalone features. This is captured in Figure 11 by the bars denoting the ‘C. T. Crunch’ dataset with different source image surfaces (see the ψ = 0° column in Figure 8). Similarly, relative to the maximum possible viewpoint invariance score, $\psi_{\delta ,\max}=\frac{1\;}{s}\left(\psi_{\max}-\psi_{\min}\right)$, has its own column in Figure 8, the proposed embedding generally scored a higher viewpoint invariance in scenes containing more surfaces, e.g., datasets observed from the NP3 camera. The highest viewpoint invariances, 74.8°, 66.15°, and 57.25°, were achieved for the ‘C. T. Crunch’, ‘H. B. Oats’, and ‘Cheez It’ datasets, respectively, which contained polygonal objects arranged from highly distinct patterns to more blurred and repeated texture patterns. Overall, the proposed embedding improved the viewpoint invariance of the representative local image features in all datasets and cameras; the proposed approach had an average viewpoint invariance of 56.9°, compared with 29.5° in the standalone case.

**Figure 12:**
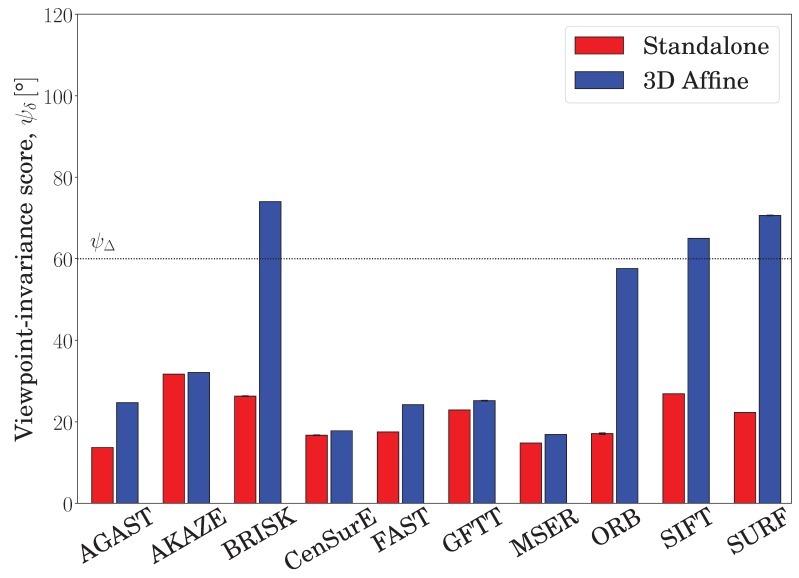
Viewpoint invariance for each detector intermixed with the SIFT [19] descriptor. Ten different detectors were evaluated. The three that exceed the targeted viewpoint invariance, $\psi_{\Delta }$, as denoted by the dotted horizontal line, were wrapped in the proposed embedding, represented by the blue bars. On the other hand, none of the standalone features approached the goal, as their red bars indicate. This figure utilizes the data from the ‘C. T. Crunch’ dataset observed from the NP3 camera. More extensive results are presented in later parts of Section 5.1.2.

Second, detectors generally benefited from the proposed embedding, with those featuring abundant and large area keypoints gaining the most benefit. To illustrate this, Figure 12 summarizes the viewpoint invariance of the detectors with the ‘C. T. Crunch’ dataset and the NP3 camera (more extensive results are reported later in this section). Although none of the standalone detectors achieved the targeted viewpoint invariance, $\psi_{\Delta }$, denoted by the dotted horizontal line, three of them (BRISK, SURF, and SIFT) achieved the target when wrapped in the proposed embedding. Furthermore, the ORB detector was only 2.4° off the target. Although MSER produces large keypoint radii, both MSER and CenSurE only had a few keypoints, which affected the correspondence establishment in both the standalone and proposed embedding results. Accordingly, MSER and CenSurE remained below the targeted viewpoint invariance, but their embedded features still outscored the standalone ones.

The highest viewpoint invariances, 74.8°, 66.15°, and 57.25°, were obtained with the BRISK, SURF, SIFT, and ORB detectors, which are characterized by a relatively high number of keypoints with medium-to-large radii. The detectors benefiting the most from the proposed embedding were ORB and SURF, which have large keypoint radii. Overall, as detailed later in this section, the standalone detectors intermixed with the SIFT descriptor scored on all datasets an average viewpoint invariance of 33.6°, which increased to 46.3° when wrapped in the proposed embedding. Detectors with abundant and considerably large keypoints benefited the most from being embedded in our method; however, for detectors with a small number of keypoints, the embedding was inadequate.

**Figure 13:**
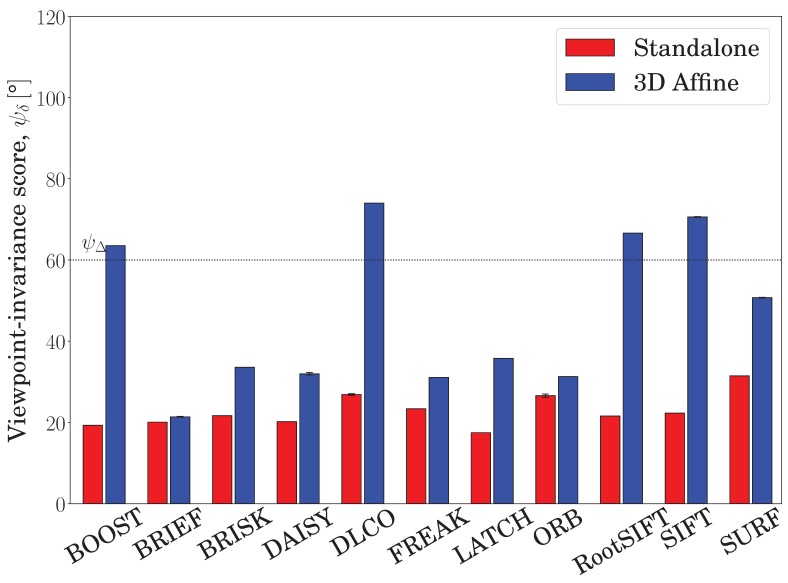
Viewpoint invariance for each descriptor intermixed with the SURF [20] detector. Eleven different descriptors were evaluated, with four of them achieving and one closely approaching the targeted viewpoint invariance threshold, $\psi_{\Delta }$, after being wrapped in the proposed embedding (blue bars). On the other hand, all standalone descriptors (red bars) missed the target. This figure utilizes the data from the ‘C. T. Crunch’ dataset and the NP3 camera. The rest of the data is presented in later parts of Section 5.1.2.

For a third study, we analyzed individual descriptors evaluated on keypoints detected by the SURF detector, as reported in Figure 13. In this experiment, all descriptors benefited from the embedding wrapping, where 4 of 11 achieved the targeted viewpoint invariance, $\psi_{\Delta }$. The highest viewpoint invariances, 74.0°, 70.6°, 65.0°, and 57.6°, were attained by DLCO, SIFT, RootSIFT, and BOOST descriptors, respectively. The descriptors benefiting the most from the proposed embedding were BOOST, SIFT, and RootSIFT. Although studying each detector invariance formula is out of scope of this manuscript, these results are most likely affected by such formulas. Overall, the average viewpoint invariance of the standalone descriptors on all datasets was 32.7°, which increased to 45.7° when wrapped in the proposed embedding.

More comprehensively, Table 3 reports the viewpoint invariance scores, $\psi_{\delta }$, for 20 pairs of various detector/descriptor with nine different setups of the four evaluation datasets and the two cameras. Out of 140 combinations with distinct surface discontinuities, the proposed embedding achieved the targeted viewpoint invariance, $\psi_{\Delta }$, in 73 combinations using 19 embedded pairs out of the total 20 pairs. On the other hand, the standalone features met the target score in only two combinations using a single pair consisting of the GFTT detector and the SIFT descriptor. These results confirm the generality of the proposed approach, in which almost all the evaluated detector/descriptor pairs gained a performance boost and achieved the targeted viewpoint invariance. Despite the first three datasets belonging to the same category of polygonal objects, the illumination, texture patterns, and contrast played an important role in establishing the difficulty of each dataset. Furthermore, with more distinct surfaces in the source image, a higher score was achieved. In light of this, ‘Pringles’ was the most challenging dataset (see the last paragraph in this section) because of its indistinct surface discontinuities, and thus no features were able to achieve the target score, in either the standalone or embedded case. Averaging over all of the datasets, the local image features scored 45.4° when embedded in the proposed framework compared with 33.3° in the standalone case. By limiting the scope to distinct surface discontinuities, the average viewpoint invariance of 37° for standalone approaches is boosted to 52.8° when wrapped in the proposed embedding.

**Figure 14:**
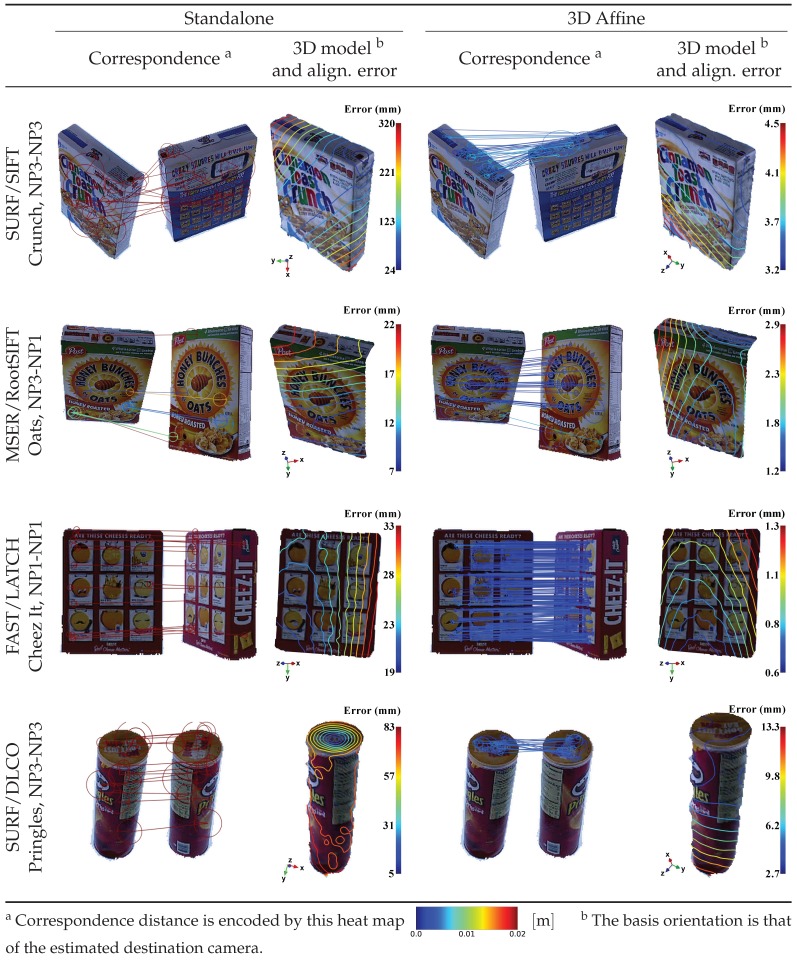
Sample qualitative cases of correspondences and 6D pose estimation using local image features, for the standalone and embedded (3D Affine) methods, under a wide viewpoint change. The alignment error, ℓ (ψ ≈ 60°), is visualized over the 3D model of the source image.

Additionally, Figure 14 shows four sample qualitative cases. The first case has a relatively small common surface whose colors are similar to the other irrelevant surfaces, thereby causing the standalone SURF/SIFT to fail miserably. On the other hand, after wrapping the same detector/descriptor pair into the proposed embedding, the alignment error, ℓ (60°), reduced from 319.6 mm to 4.5 mm. The second case involved a viewpoint change in both azimuth and elevation directions, with less colorful surfaces and different illumination conditions, which reduced the inlier ratio. Despite this, the proposed embedding handled the case properly. In the third case, similar repeated patterns resulted in many outliers; however, again, the proposed embedding recovered the correct correspondence with abundant inliers and an alignment error of only ℓ (60°) = 1.4 mm. The fourth case has a cylindrical surface, which is challenging for the labeling process (Section 2.2) and has a high outlier ratio. The proposed approach using an embedded local image feature still outperformed the standalone one by using the top surfaces to recover a good correspondence.

**Figure 15:**
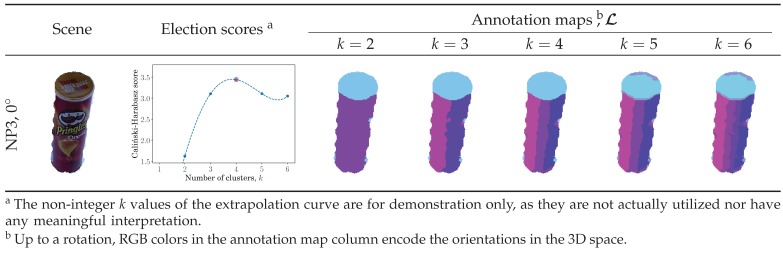
Although approximating curved surfaces as planar segments improves the viewpoint representation, some interesting points are split apart, which drastically affects their descritability.

Although the proposed embedding had the lowest performance on the ‘Pringles’ dataset (Table 3), such a bottleneck is only due to the smooth-surface labeling phase (Section 2.2). As illustrated in Figure 15, the indistinct surface discontinuities cause the cylindrical surface to be segmented into thin longitudinal strips (k≥3 columns) that split some local regions, thus reducing their describability. Furthermore, connectivity issues are observed, in which non-uniform longitudinal strips (e.g., k={3,6} columns) are formed, and some tiny fragments at the boundaries are scattered away from their corresponding clusters. Based on these observations, constraining the clusters by smoothness [62], incorporating *k*-NN connectivity [63], and performing non-rigid registration [64] of the curved surface into the virtual image plane, will all improve the overall performance, individually or collectively. Moreover, since only surface normals are employed in the label computation, it is also expected that employing depth can improve the results.

### 5.2. Sensitivity Analysis Experiments

So far, we have reported several experiments conducted in ideal and realistic situations (Section 5.1), and the results demonstrate both effectiveness and generality of our proposal. In this section, we demonstrate the robustness of the proposed method to depth noise. Different SNRs were considered in a synthetic setup. Additionally, while it is sufficient to utilize one dataset, two datasets (resembling the second and third columns in Figure 8) were evaluated to avoid any possible bias.

The total of 90 matching experiments, as reported in Section 5.1.1, constitute just 5% of this experiment. The ideal setup experiments were performed on the synthetic ‘C. T. Crunch (NP1 - v2)’ dataset. The results of the noise-free experiment are viewpoint invariances of 75.89° and 113.53° for the standalone and proposed approaches, respectively, which corresponds to *SNR* = 65 dB. As a reference, the scores obtained at *SNR* = 65 dB of the standalone approach are kept constant throughout the entire range, even though their scores actually degraded with the depth noise since one needs to compute the keypoint 3D coordinates (refer to Section 3.2 for details).

**Figure 16:**
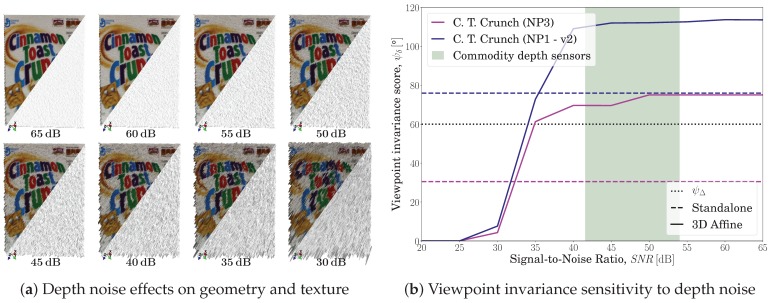
(**a**) a demonstration of different tested noise levels. The frame shows one of the 140 synthetic frames utilized, which simulated the ‘C. T. Crunch’ dataset with different viewpoints and cameras (the first two rows of Figure 8); (**b**) the proposed embedding achieved the targeted viewpoint invariance, $\psi_{\Delta }$, for a wide range of depth noise, *SNR* ≥ 35 dB, in both datasets, and it outperformed the standalone intensity-based approach in estimating viewpoint rotations despite the ideal intensity setting, in which no illumination or blur effects were synthesized.

Figure 16 shows the proposed approach response to different SNR levels. To clearly demonstrate the evaluated noise levels, we show in Figure 16a a sample RGB-D frame projected into 3D with different noise levels. To better exhibit the noise effects, the frame is observed from a different viewpoint than that of its camera. It is worth noting that, even under ideal situations, some tiny protuberances can be seen on the geometry (the lower-triangle part), but it is only at high noise levels that our eyes notice any texture defects.

The proposed method generates a proportional response to different SNRs and demonstrates robustness to noise starting from 35 dB and higher, as shown in Figure 16b. At around the same *SNR* = 35 dB value, the proposed embedding outperformed the standalone approach in both datasets while also achieving the targeted viewpoint invariance, $\psi_{\Delta }$. Starting around 40 dB–50 dB, the curves approach a saturation value, which is related to the dataset-dependent maximum viewpoint invariance score (denoted by the $\psi_{\psi ,\max}$ in Figure 8). Knowing that low-cost commodity depth sensors exhibit 50 dB–54 dB within the first meter and subsequently deteriorates quadratically with depth to 41.58 dB–46.62 dB at a 3 m depth [65,66], it is safe to conclude that our proposed approach can operate robustly in the presence of input noise, and well beyond. Accordingly, the proposed embedding might even operate without requiring an external depth-map input; instead, it may compute the depth maps fromthe RGB image sequences using approaches like DTAM(Dense Tracking AndMapping) [67], despite any potential high noise levels. This provides an interesting point worthy of further investigation.

## 6. Conclusions

Despite their variety, local image features suffer from a lack of robustness to viewpoint changes. Improving the viewpoint invariance has compelling applications in different fields and is gaining increasing attention, especially with the availability of geometric information attained through depth sensors. In this manuscript, we propose a general embedding framework that empowers standalone local image features with viewpoint invariance in a generic manner, without depending on a specific local image feature type or requiring interface or internal adaptations. The proposed embedding utilizes depth information to annotate smooth surfaces and then warps the annotated surfaces to a viewpoint invariant representation, wherein all the feature-related computations take place. A nonparametric labeling of smooth surfaces is proposed to achieve robust annotation of distinct surfaces by clustering surface normals using the spherical *k*-means algorithm, where *k* is determined by the Caliński–Harabasz score. Invariant warping is achieved using a hybrid rigid–homography method, where a good balance between quality and accuracy is maintained, thereby improving the repeatability and distinctiveness of the detected and extracted features. Keypoints are then detected, and descriptors are computed from the viewpoint invariant representation, followed by keypoint remapping to the input space. The proposed embedding increased the stability against viewpoint changes (i.e., *viewpoint invariance*) by leveraging the geometric information of a given scene. The performance was evaluated quantitatively by measuring the accumulative length of the relative viewpoint axis having tolerated error.

Initially, a brief comparison in an ideal setup was conducted between the proposed embedding and the highly respected SIFT/SURF detector and descriptor. The standalone density-based approach could not keep up with large out-of-plane rotations, and it turned out that both the detector repeatability and the descriptor distinctiveness are affected by viewpoint changes. While the detector repeatability was not severely affected, the descriptors face much greater difficulty in computing distinct descriptions for physically identical keypoints observed from different viewpoints. Most of the issue lies in the sampling-window size, which has no information about the underlying geometry. In this case, the best strategy, i.e., decreasing the keypoint area, also fails. On the contrary, the proposed method facilitated more repeatable detection and more distinctive description with its viewpoint-independent surface representation.

Furthermore, empirical results on real-world datasets showed that various detectors and descriptors, regardless of their invariance formula(s) or implementation, benefited from the proposed embedding. This was tested on several datasets to varying extents, and depended on the keypoint size and object geometry. For keypoint size, the proposed embedding best contributes to the increased viewpoint invariance of detectors that have abundant and considerably large keypoints. We evaluated ten different local image detectors intermixed with the SIFT descriptor, and they obtained an average viewpoint invariance of 46.3° when wrapped in the proposed embedding, compared with 33.6° in the standalone case. Similarly, 11 different local image descriptors were intermixed with the SURF detector, and they achieved an overall average viewpoint invariance of 45.7° when embedded in the proposed framework, as compared with 32.7° in the standalone case.

For object geometry, the best results achieved were with polygonal objects, whose surface discontinuities are more distinct. Furthermore, objects with bright colors and distinct patterns were associated with higher scores in the proposed embedding than those obtained with blurrier and repeated-pattern objects. Out of 140 test cases involving 20 detector/descriptor pairs and distinct-surface discontinuities, the proposed embedding scored an average viewpoint invariance of 52.8° and achieved the targeted viewpoint invariance, $\psi_{\Delta }$, in 73 cases belonging to 19 pairs as compared with an average of 37° and only two cases reaching the target, $\psi_{\Delta }$, both belonging to a single standalone feature pair. Our embedding demonstrated some generality, based on the fact that 19 out of 20 local image features benefited from it, and scored the target invariance in several cases, while only one standalone local image feature managed to score in two test cases. Overall, standalone features had an average viewpoint invariance of 33.3° on all evaluated datasets, which improved to 45.4° with the proposed embedding. In terms of noise sensitivity, the proposed method proved tolerant to noise beyond the requirements of 41.58 dB imposed by low-cost commodity depth sensors within the first 3 m of depth, as it can withstand SNRs as low as 35 dB.

This study addresses keypoint detection and feature computation under a wide baseline and demonstrates a large viewpoint invariance gain in cases of distinct surface discontinuities. On the other hand, the proposed embedding was unable to increase the viewpoint invariance for detectors of few keypoints or objects of complex geometries and indistinct surface-normal discontinuities. This limitation stems from the smooth-surface labeling phase, in which the spherical *k*-means utilizes only the surface normals to label different surfaces. To overcome this limitation, we consider the possibility of including depth discontinuities, constraining the clusters by *k*-NN pixel-based connectivity [63], imposing smoothness [62] constraints, or performing non-rigid registration [64] of the surface after the rigid alignment to the virtual image plane. Furthermore, there remains room for improvement in regard to the current hybrid rigid–homography method, which depends on the homography transforms, where index interpolation with iterative back-projection [68] represents another alternative and constitutes a point of future work. Currently, depth input is needed, which is a reasonable requirement given the prevalence of low-cost commodity depth sensors nowadays. However, since the proposed method can handle low SNRs, we find computing depth maps from RGB image sequences using approaches like DTAM [67] to be an interesting extension of this research. Similarly, depth maps are currently interpolated for missing-depth small regions while neglecting larger ones; incorporating gradient-aware depth painting/interpolation constitutes a potential future direction.

Various applications, including 6D pose estimation, will be able to benefit from the proposed embedding beyond what was demonstrated in this manuscript, by using normal and gradient orientations of the region-affine keypoints along with their center points (refer to Section 3.2 for details). It is also believed that the current work can be utilized in non-rigid environments by replacing RANSAC-based matching with a global-motion modeling technique, such as factorized graph matching [69]. Additionally, fine alignment can be implemented by using iterative closest/corresponding point variants (color [70] or point-to-plane [71]). Global alignment with sensor-uncertainty modeling using sparse-surface adjustment [72] appears to improve high-level performance, especially when considering multi-way alignment. Ultimately, we plan to adapt this work to a 3D-reconstruction application. 

## Figures and Tables

**Table 1:**
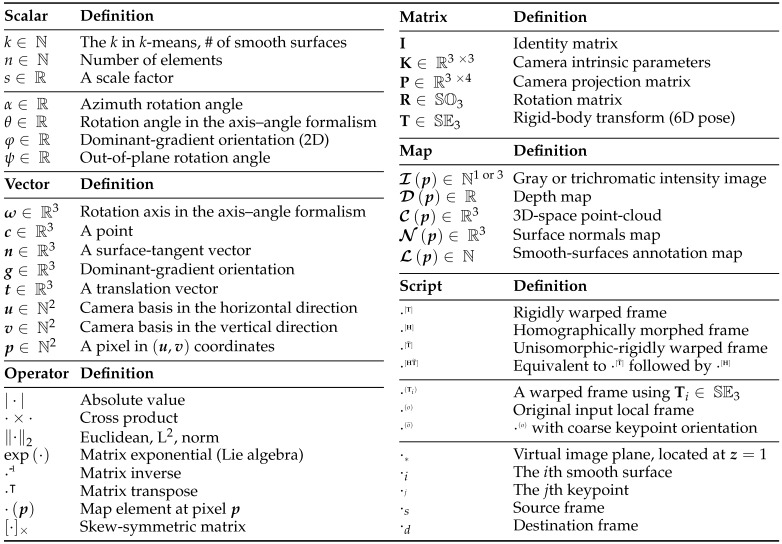
Table of notations used throughout the manuscript, where N is the natural numbers set, R is the real numbers set, SO is the special orthogonal group, and SE is the special Euclidean group.

**Table 2:** List of 10 detectors and 11 descriptors against which the proposed embedding was evaluated. For practicality, all detectors were intermixed with a SIFT [19] descriptor, and all descriptors were intermixed with a SURF [20] detector.

Local Image Feature	Detector	Descriptor
AGAST (Adaptive & Generic detection based on Accelerated Segment Test) [33]	**✓**	—
AKAZE (Accelerated KAZE) [38]	**✓**	—
BOOST (Named after a utilized machine learning concept; the boosting) [6]	—	**✓**
BRIEF (Binary Robust Independent Elementary Features) [32]	—	**✓**
BRISK (Binary Robust Invariant Scalable Keypoints) [35]	**✓**	**✓**
CenSurE (Center Surround Extremas detector) [28]	**✓**	—
DAISY (Named after a flower resembling its sampling window) [31]	—	**✓**
DLCO (Descriptor Learning using Convex Optimization) [5]	—	**✓**
FAST (Features from Accelerated Segment Test) [30]	**✓**	—
FREAK (Fast Retina Keypoint) [37]	—	**✓**
GFTT (Good Features to Track) [18]	**✓**	—
LATCH (Learned Arrangements of Three Patch Codes) [39]	—	**✓**
MSER (Maximally Stable Extremal Regions) [29]	**✓**	—
ORB (Oriented FAST and Rotated BRIEF) [34]	**✓**	**✓**
RootSIFT (Root-normalized SIFT descriptor) [36]	—	**✓**
SIFT (Scale-Invariant Feature Transform) [19]	**✓**	**✓**
SURF (Speeded Up Robust Features) [20]	**✓**	**✓**

**Table 3:** Overall viewpoint invariance, $\psi_{\delta }$, for various features and datasets. The scores exceeding the targeted threshold, $\psi_{\Delta }$, are shown in bold. Detectors and descriptors are detailed in Table 2.

	Detect./Desc.	C. T. Crunch			H. B. Oats		Cheez It		Pringles		Average
		NP3	NP1 - v2	NP1	NP3	NP1	NP3	NP1	NP3	NP1	
**Standalone**	AGAST/SIFT	13.7° ± 0.0°	55.2° ± 0.0°	56.5° ± 0.0°	20.3° ± 0.0°	56.1° ± 0.0°	12.7° ± 0.0°	54.9° ± 0.0°	15.7° ± 0.0°	24.7° ± 0.0°	34.4° ± 0.0°
	AKAZE/SIFT	31.7° ± 0.0°	52.7° ± 0.0°	55.1° ± 0.0°	29.2° ± 0.0°	53.2° ± 0.0°	6.2° ± 0.0°	47.5° ± 0.0°	15.4° ± 0.0°	25.0° ± 0.0°	35.1° ± 0.0°
	BRISK/SIFT	26.3° ± 0.1°	51.9° ± 0.0°	53.9° ± 0.1°	39.1° ± 0.3°	54.1° ± 0.0°	15.0° ± 0.0°	50.5° ± 0.1°	17.1° ± 0.0°	24.2° ± 0.0°	36.9° ± 0.1°
	CenSurE/SIFT	16.7° ± 0.1°	53.3° ± 0.0°	56.7° ± 0.0°	14.1° ± 0.0°	54.7° ± 0.0°	13.7° ± 0.0°	54.6° ± 0.0°	1.9° ± 0.0°	18.2° ± 0.1°	31.5° ± 0.0°
	FAST/SIFT	17.5° ± 0.0°	55.8° ± 0.0°	55.6° ± 0.0°	20.7° ± 0.0°	55.2° ± 0.0°	18.8° ± 0.0°	55.9° ± 0.0°	15.8° ± 0.0°	24.3° ± 0.0°	35.5° ± 0.0°
	GFTT/SIFT	22.9° ± 0.0°	**77.7° ± 0.1°**	59.2° ± 0.1°	25.6° ± 0.9°	**60.5° ± 0.0°**	19.6° ± 0.0°	57.9° ± 0.0°	19.1° ± 0.0°	40.3° ± 0.0°	42.5° ± 0.3°
	MSER/SIFT	14.8° ± 0.0°	40.7° ± 0.3°	50.2° ± 0.0°	18.2° ± 0.0°	43.6° ± 0.0°	0.0° ± 0.0°	31.3° ± 0.0°	8.8° ± 0.0°	21.7° ± 0.3°	25.5° ± 0.2°
	ORB/SIFT	17.1° ± 0.2°	37.9° ± 0.0°	52.0° ± 0.0°	23.1° ± 0.0°	45.8° ± 0.0°	12.3° ± 0.0°	44.4° ± 0.0°	16.5° ± 0.0°	23.8° ± 0.0°	30.3° ± 0.1°
	SIFT	26.9° ± 0.0°	53.4° ± 0.0°	55.3° ± 0.0°	31.0° ± 0.0°	53.1° ± 0.1°	0.0° ± 0.0°	52.2° ± 0.0°	21.2° ± 0.1°	18.4° ± 0.0°	34.6° ± 0.0°
	SURF	31.5° ± 0.0°	52.9° ± 0.4°	51.9° ± 0.0°	28.3° ± 0.0°	56.6° ± 0.0°	19.0° ± 0.0°	49.3° ± 0.0°	17.7° ± 0.0°	27.9° ± 0.0°	37.2° ± 0.1°
	SURF/BOOST	19.3° ± 0.0°	49.2° ± 0.1°	48.1° ± 0.1°	20.5° ± 0.0°	47.4° ± 0.0°	6.3° ± 0.0°	39.6° ± 0.0°	15.7° ± 0.0°	24.3° ± 0.0°	30.0° ± 0.0°
	SURF/BRIEF	20.1° ± 0.0°	52.2° ± 0.0°	55.5° ± 0.2°	23.3° ± 0.0°	54.4° ± 0.2°	7.8° ± 0.0°	55.8° ± 0.0°	16.4° ± 0.0°	24.3° ± 0.0°	34.4° ± 0.1°
	SURF/BRISK	21.7° ± 0.0°	50.4° ± 0.0°	48.7° ± 0.0°	23.3° ± 0.1°	50.9° ± 0.1°	3.5° ± 0.0°	49.3° ± 0.0°	17.0° ± 0.0°	23.7° ± 0.0°	32.1° ± 0.0°
	SURF/DAISY	20.2° ± 0.0°	56.3° ± 0.0°	56.1° ± 0.0°	26.0° ± 0.0°	57.1° ± 0.0°	22.8° ± 0.0°	50.1° ± 0.0°	17.3° ± 0.0°	24.5° ± 0.0°	36.7° ± 0.0°
	SURF/DLCO	26.9° ± 0.2°	52.8° ± 0.0°	50.9° ± 0.0°	28.3° ± 0.0°	52.8° ± 0.0°	8.8° ± 0.0°	40.5° ± 0.0°	16.4° ± 0.0°	23.9° ± 0.0°	33.5° ± 0.1°
	SURF/FREAK	23.4° ± 0.0°	46.7° ± 0.2°	49.8° ± 0.1°	23.7° ± 0.0°	45.1° ± 0.0°	4.4° ± 0.0°	47.6° ± 0.0°	18.1° ± 0.0°	24.3° ± 0.0°	31.5° ± 0.1°
	SURF/LATCH	17.5° ± 0.0°	48.7° ± 0.0°	48.5° ± 0.0°	16.8° ± 0.0°	47.9° ± 0.1°	12.8° ± 0.0°	42.0° ± 0.0°	16.5° ± 0.0°	24.1° ± 0.0°	30.5° ± 0.0°
	SURF/ORB	26.6° ± 0.4°	50.9° ± 0.0°	49.4° ± 0.0°	24.5° ± 0.2°	51.7° ± 0.0°	21.4° ± 0.0°	46.0° ± 0.0°	18.0° ± 0.0°	29.6° ± 0.0°	35.3° ± 0.1°
	SURF/RootSIFT	21.6° ± 0.0°	44.2° ± 0.0°	46.7° ± 0.0°	24.0° ± 0.0°	46.1° ± 0.0°	6.0° ± 0.0°	28.9° ± 0.0°	15.7° ± 0.0°	24.2° ± 0.0°	28.6° ± 0.0°
	SURF/SIFT	22.3° ± 0.0°	45.9° ± 0.3°	49.2° ± 0.0°	24.7° ± 0.0°	48.9° ± 0.0°	6.7° ± 0.0°	28.9° ± 0.0°	15.7° ± 0.0°	23.7° ± 0.0°	29.5° ± 0.1°
	**Average**	21.9° ± 0.1°	51.4° ± 0.1°	52.5° ± 0.1°	24.2° ± 0.2°	51.8° ± 0.0°	10.9° ± 0.0°	46.4° ± 0.0°	15.8° ± 0.0°	24.8° ± 0.1°	33.3° ± 0.1°
	ψδψδ,max **ratio**	29.2%	38.07%	58.33%	32.27%	57.56%	14.5%	51.56%	21.07%	30.61%	37.02%
**3D Affine**	AGAST/SIFT	24.7° ± 0.0°	**96.8° ± 0.0°**	**61.9° ± 0.0°**	27.0° ± 0.0°	**64.0° ± 0.0°**	22.9° ± 0.0°	**62.8° ± 0.0°**	26.6° ± 0.0°	56.3° ± 0.5°	49.2° ± 0.2°
	AKAZE/SIFT	32.1° ± 0.0°	57.9° ± 0.0°	**63.1° ± 0.0°**	31.3° ± 0.2°	**61.8° ± 0.0°**	14.2° ± 0.2°	**62.5° ± 0.0°**	11.0° ± 0.0°	0.0° ± 0.0°	37.1° ± 0.1°
	BRISK/SIFT	**74.0° ± 0.0°**	56.0° ± 0.0°	**60.1° ± 0.0°**	49.4° ± 0.0°	**60.3° ± 0.0°**	55.6° ± 0.0°	59.3° ± 0.0°	16.0° ± 0.0°	13.5° ± 0.2°	49.4° ± 0.1°
	CenSurE/SIFT	17.8° ± 0.0°	**63.8° ± 0.0°**	**66.9° ± 0.0°**	28.1° ± 0.0°	**64.3° ± 0.0°**	0.0° ± 0.0°	**67.0° ± 0.0°**	0.0° ± 0.0°	0.0° ± 0.0°	34.2° ± 0.0°
	FAST/SIFT	24.2° ± 0.0°	**97.1° ± 0.0°**	**61.7° ± 0.0°**	26.7° ± 0.0°	**63.7° ± 0.0°**	21.0° ± 0.0°	**62.7° ± 0.0°**	27.0° ± 0.0°	57.4° ± 0.0°	49.1° ± 0.0°
	GFTT/SIFT	25.2° ± 0.1°	**95.9° ± 0.0°**	**61.1° ± 0.1°**	30.4° ± 0.0°	**63.3° ± 0.0°**	25.7° ± 0.0°	**61.9° ± 0.0°**	26.9° ± 0.0°	54.4° ± 0.0°	49.4° ± 0.1°
	MSER/SIFT	16.9° ± 0.0°	55.3° ± 0.0°	57.4° ± 0.0°	25.6° ± 0.0°	54.4° ± 0.0°	0.0° ± 0.0°	59.9° ± 0.0°	5.2° ± 0.0°	11.3° ± 0.0°	31.8° ± 0.0°
	ORB/SIFT	57.6° ± 0.0°	57.7° ± 0.0°	**61.0° ± 0.0°**	41.7° ± 0.0°	**61.6° ± 0.0°**	25.0° ± 0.0°	**60.8° ± 0.0°**	13.6° ± 0.0°	30.0° ± 0.0°	45.4° ± 0.0°
	SIFT	**65.0° ± 0.0°**	**89.8° ± 0.0°**	**60.3° ± 0.0°**	**75.0° ± 0.0°**	**61.2° ± 0.0°**	**61.2° ± 0.1°**	59.4° ± 0.0°	21.5° ± 0.1°	46.9° ± 0.5°	**60.0° ± 0.2°**
	SURF	50.7° ± 0.1°	**91.5° ± 0.0°**	**61.1° ± 0.0°**	48.1° ± 0.2°	**63.0° ± 0.0°**	43.7° ± 0.7°	**60.3° ± 0.0°**	21.7° ± 0.0°	25.8° ± 0.0°	51.8° ± 0.2°
	SURF/BOOST	**63.5° ± 0.0°**	**72.8° ± 0.1°**	**61.5° ± 0.0°**	**63.8° ± 0.0°**	**60.9° ± 0.1°**	36.1° ± 0.8°	**60.8° ± 0.0°**	20.0° ± 0.0°	13.2° ± 0.8°	50.3° ± 0.4°
	SURF/BRIEF	21.4° ± 0.1°	**71.4° ± 0.0°**	**60.7° ± 0.0°**	23.6° ± 0.0°	**63.3° ± 0.0°**	14.6° ± 0.0°	**60.2° ± 0.0°**	15.2° ± 0.0°	9.9° ± 0.1°	37.8° ± 0.1°
	SURF/BRISK	33.6° ± 0.0°	55.5° ± 0.0°	**60.4° ± 0.0°**	32.7° ± 0.0°	**60.0° ± 0.0°**	0.0° ± 0.0°	**60.9° ± 0.0°**	16.0° ± 0.0°	0.0° ± 0.0°	35.5° ± 0.0°
	SURF/DAISY	32.0° ± 0.3°	**94.2° ± 0.1°**	**60.5° ± 0.0°**	30.2° ± 0.0°	**61.6° ± 0.0°**	24.4° ± 0.0°	58.9° ± 0.0°	27.9° ± 0.0°	53.6° ± 0.1°	49.3° ± 0.1°
	SURF/DLCO	**74.0° ± 0.0°**	**94.0° ± 0.0°**	**61.5° ± 0.0°**	**64.5° ± 0.0°**	**62.8° ± 0.0°**	46.0° ± 0.0°	59.3° ± 0.1°	20.5° ± 0.0°	16.4° ± 0.0°	55.4° ± 0.0°
	SURF/FREAK	31.1° ± 0.0°	**62.7° ± 0.0°**	**68.5° ± 0.0°**	30.0° ± 0.0°	**68.0° ± 0.0°**	0.0° ± 0.0°	**63.5° ± 0.0°**	1.5° ± 0.0°	0.0° ± 0.0°	36.1° ± 0.0°
	SURF/LATCH	35.8° ± 0.0°	56.6° ± 0.0°	**60.1° ± 0.0°**	38.7° ± 0.1°	**60.0° ± 0.0°**	18.2° ± 0.0°	60.0° ± 0.0°	19.1° ± 0.0°	1.8° ± 0.0°	38.9° ± 0.0°
	SURF/ORB	31.3° ± 0.0°	55.6° ± 0.0°	**61.8° ± 0.0°**	36.4° ± 0.0°	**61.7° ± 0.0°**	5.6° ± 0.2°	**60.3° ± 0.0°**	17.9° ± 0.1°	5.5° ± 0.0°	37.3° ± 0.1°
	SURF/RootSIFT	**66.6° ± 0.0°**	**81.7° ± 0.0°**	**60.3° ± 0.0°**	55.7° ± 0.0°	59.8° ± 0.0°	59.4° ± 0.0°	59.7° ± 0.0°	20.9° ± 0.0°	12.5° ± 0.2°	53.0° ± 0.1°
	SURF/SIFT	**70.6° ± 0.1°**	**93.5° ± 0.0°**	**60.3° ± 0.0°**	**71.7° ± 0.0°**	**60.6° ± 0.1°**	55.1° ± 0.0°	59.4° ± 0.0°	21.4° ± 0.0°	19.1° ± 0.1°	56.9° ± 0.0°
	**Average**	42.4° ± 0.1°	**75.0° ± 0.0°**	**61.5° ± 0.0°**	41.5° ± 0.1°	**61.8° ± 0.0°**	26.4° ± 0.2°	**61.0° ± 0.0°**	17.5° ± 0.0°	21.4° ± 0.2°	45.4° ± 0.1°
	ψδψδ,max **ratio**	56.53%	55.56%	68.33%	55.33%	68.67%	35.20%	67.78%	23.33%	26.42%	50.79%

## Abbreviations

The following abbreviations are used in this manuscript:

*k*-NN	*k*-Nearest Neighbor
6D	A short form of 6 DoFs (Degrees of Freedom)
AGAST	Adaptive & Generic detection based on Accelerated Segment Test
AKAZE	Accelerated KAZE
ASIFT	2D Affine SIFT
ASURF	2D Affine SURF
BOOST	Named after a utilized machine learning concept; the boosting
BRIEF	Binary Robust Independent Elementary Features
BRISK	Binary Robust Invariant Scalable Keypoints
CenSurE	Center Surround Extremas detector
DAISY	Named after a flower resembling its sampling window
DLCO	Descriptor Learning using Convex Optimization
DoF	Degree of Freedom
DTAM	Dense Tracking And Mapping
FAST	Features from Accelerated Segment Test
FREAK	Fast Retina Keypoint
GFTT	Good Features to Track
KAZE	Named after the “wind” in Japanese; an analogy to nonlinear diffusion
LATCH	Learned Arrangements of Three Patch Codes
MSER	Maximally Stable Extremal Regions
ORB	Oriented FAST and Rotated BRIEF
RANSAC	Random Sample Consensus
RGB-D	RGB trichromatic color image and per-pixel Depth map
RMS	Root-Mean-Square
RootSIFT	Root-normalized SIFT descriptor
SIFT	Scale-Invariant Feature Transform
SLAM	Simultaneous Localization And Mapping
SNR	Signal-to-Noise Ratio
SURF	Speeded Up Robust Features
SVD	Singular Value Decomposition
VIP	Viewpoint Invariant Patch
